# Structural dissection of two redox proteins from the shipworm symbiont *Teredinibacter turnerae*

**DOI:** 10.1107/S2052252524001386

**Published:** 2024-03-01

**Authors:** Badri S. Rajagopal, Nick Yates, Jake Smith, Alessandro Paradisi, Catherine Tétard-Jones, William G. T. Willats, Susan Marcus, J. Paul Knox, Mohd Firdaus-Raih, Bernard Henrissat, Gideon J. Davies, Paul H. Walton, Alison Parkin, Glyn R. Hemsworth

**Affiliations:** a Astbury Centre for Structural Molecular Biology and School of Molecular and Cellular Biology, Faculty of Biological Sciences, University of Leeds, Leeds LS2 9JT, United Kingdom; b Department of Chemistry, University of York, York YO10 5DD, United Kingdom; c School of Natural and Environmental Science, Newcastle University, Newcastle upon Tyne NE1 7RU, United Kingdom; d Centre for Plant Sciences, Faculty of Biological Sciences, University of Leeds, Leeds LS2 9JT, United Kingdom; e Department of Applied Physics, Faculty of Science and Technology, Universiti Kebangsaan Malaysia, 43600 UKM Bangi, Malaysia; f Architecture et Fonction des Macromolécules Biologiques (AFMB), CNRS, Aix-Marseille Université, Marseille, France; g INRA, USC 1408 AFMB, Marseille, France; h Department of Biological Sciences, King Abdulaziz University, Jeddah, Saudi Arabia; Chinese Academy of Sciences, China

**Keywords:** shipworms, cellulose, redox proteins, lytic polysaccharide monooxygenases, *c*-type cytochromes, electron transfers, protein structures, X-ray crystallography, *Teredinibacter turnerae*

## Abstract

The discovery of lytic polysaccharide monooxygenases (LPMOs), a family of copper-dependent enzymes that play a major role in polysaccharide degradation, has revealed the importance of oxidoreductases in the biological utilization of biomass. In fungi, a range of redox proteins have been implicated as working in harness with LPMOs to bring about polysaccharide oxidation. In bacteria, less is known about the interplay between redox proteins and LPMOs, or how the interaction between the two contributes to polysaccharide degradation. We therefore set out to characterize two previously unstudied proteins from the shipworm symbiont *Teredinibacter turnerae* that were initially identified by the presence of carbohydrate binding domains appended to uncharacterized domains with probable redox functions. Here, X-ray crystal structures of several domains from these proteins are presented together with initial efforts to characterize their functions. The analysis suggests that the target proteins are unlikely to function as LPMO electron donors, raising new questions as to the potential redox functions that these large extracellular multi-haem-containing *c*-type cytochromes may perform in these bacteria.

## Abstract 2

The structures of novel redox domains extracted from two large extracellular redox proteins encoded in the genome of *Teredinibacter turnerae* suggest potentially novel roles for electron-transfer proteins in carbohydrate chemistry outside of the cell.

## 1. Introduction

Lytic polysaccharide monooxygenases (LPMOs) are a set of enzymes that have been at the centre of attention for over a decade due to their ability to enhance the efficiency of enzymatic cellulose degradation [see Beeson *et al.* (2015 ▸), Ciano *et al.* (2018 ▸), Hemsworth *et al.* (2015 ▸) and Vaaje-Kolstad *et al.* (2017 ▸) for extensive recent reviews]. These copper-dependent enzymes, using an electron source and either O_2_ or H_2_O_2_, catalyse the site-specific addition of a single oxygen atom at either the C1 or C4 position of the glucose ring within cellulose (and other polysaccharide) chains. This modification destabilizes the 1,4 glycosidic linkage and thereby brings about oxidative polysaccharide cleavage (Vaaje-Kolstad *et al.*, 2010 ▸; Quinlan *et al.*, 2011 ▸; Phillips *et al.*, 2011 ▸; Bissaro *et al.*, 2017 ▸), and can boost the ability of other canonical glycoside hydro­lases to further degrade the substrate. This boosting action leads to significant increases in the level of glucose that can be obtained from lignocellulosic biomass for processing into bio­ethanol (Vaaje-Kolstad *et al.*, 2010 ▸; Cannella *et al.*, 2012 ▸; Harris *et al.*, 2010 ▸; Lo Leggio *et al.*, 2015 ▸; Sabbadin, Hemsworth *et al.*, 2018 ▸), and more recently, new biological roles for LPMOs have also emerged, with these enzymes being implicated in virulence (Askarian *et al.*, 2021 ▸, 2023 ▸; Sabbadin *et al.*, 2021 ▸), copper homeostasis (Garcia-Santamarina *et al.*, 2020 ▸) and cell-wall remodelling (Zhong *et al.*, 2022 ▸).

When O_2_ is the co-substrate, LPMOs require a source of electrons to allow them to function. In this regard, LPMOs are known to use a range of electron sources including small-molecule reducing agents (Vaaje-Kolstad *et al.*, 2010 ▸; Beeson *et al.*, 2012 ▸; Couturier *et al.*, 2018 ▸, Filiatrault-Chastel *et al.*, 2019 ▸; Hemsworth *et al.*, 2014 ▸; Isaksen *et al.*, 2014 ▸; Lo Leggio *et al.*, 2015 ▸; Phillips *et al.*, 2011 ▸; Quinlan *et al.*, 2011 ▸; Sabbadin, Hemsworth *et al.*, 2018 ▸; Vu *et al.*, 2014 ▸; Simmons *et al.*, 2017 ▸), phenolic compounds derived from lignin or chitin (Kracher *et al.*, 2016 ▸; Dimarogona *et al.*, 2012 ▸; Garajova *et al.*, 2016 ▸; Kommedal *et al.*, 2022 ▸) and even chloro­phyll, which allows the reaction to be driven by light (Cannella *et al.*, 2016 ▸). In fungi, cellobiose de­hydrogenase (CDH) (Phillips *et al.*, 2011 ▸; Sygmund *et al.*, 2012 ▸), other members of the glucose–methanol–choline (GMC) oxidoreductase family of enzymes (Garajova *et al.*, 2016 ▸; Kracher *et al.*, 2016 ▸) and AA12 pyrrolo­quinoline quinone (PQQ)-dependent pyran­ose de­hydrogenases (Várnai *et al.*, 2018 ▸) have also been demonstrated as capable LPMO electron donors. CDH is the best known of these enzymes and has a two-domain architecture consisting of a large catalytic domain connected via a flexible linker to a small *b*-type cytochrome domain (Tan *et al.*, 2015 ▸; Zamocky *et al.*, 2006 ▸). The catalytic domain harbours a single molecule of flavin adenine dinucleotide (FAD), which catalyses the oxidation of cellobiose to cellobiono-δ-lactone generating FADH_2_. The electrons liberated by this reaction can then be used to generate H_2_O_2_ (Bao *et al.*, 1993 ▸; Pricelius *et al.*, 2009 ▸; Wilson *et al.*, 1990 ▸) or can be passed via the *b*-type cytochrome domain to an LPMO thereby reducing the active-site copper for activity (Kracher *et al.*, 2016 ▸; Phillips *et al.*, 2011 ▸; Sygmund *et al.*, 2012 ▸). Recently, much of the focus on LPMOs has shifted since some reactions have been demonstrated to be initially more rapid when the enzymes are provided with H_2_O_2_ rather than O_2_ (Bissaro *et al.*, 2017 ▸, 2020 ▸; Kont *et al.*, 2020 ▸; Kuusk *et al.*, 2018 ▸, 2019 ▸). How the other enzymes that have been implicated in LPMO biochemistry fit into such a reaction coordinate requires further investigation (Hedison *et al.*, 2021 ▸), and indeed there remains much to be explored in terms of the availability of the reaction components present in the natural environment [see Hemsworth (2023 ▸) for a recent review].

Whilst much of the research on LPMOs has focused on fungal systems, bacteria also make significant use of LPMOs in their cellulose and chitin degradation machinery (Horn *et al.*, 2012 ▸; Vaaje-Kolstad *et al.*, 2013 ▸). In contrast to fungi, little is known about the roles of other redox enzymes in these processes in bacteria, not least because an enzyme capable of playing a similar role to CDH has yet to be identified in this kingdom of life. Cbp2D and Cbp2E in *Cellvibrio japonicus* are the only proteins that have been suggested as potential proteinaceous LPMO redox partners from bacteria, to our knowledge (Gardner *et al.*, 2014 ▸). Knockout of the genes encoding these proteins leads to a significant reduction in *C. japonicus*’ ability to metabolize cellulose, implying an important role for them during cellulose deconstruction (Gardner *et al.*, 2014 ▸). Cbp2D and Cbp2E both harbour domains that may contain redox cofactors (termed X158, X183 and X132 in subsequent sections) appended to domains that assist in enzymes binding to polysaccharides – carbohydrate binding modules (CBMs) (Branch *et al.*, 2021 ▸; Gardner *et al.*, 2014 ▸; Vincent *et al.*, 2010 ▸). Our previous analysis demonstrated that an X183 domain from Cbp2D was capable of donating electrons to an LPMO for activity, albeit slowly (Branch *et al.*, 2021 ▸), and so we set out to investigate other proteins with similarities to Cbp2D.

Another bacterial species that encodes a plethora of carbohydrate-active enzymes is *Teredinibacter turnerae*, which is best known for its role as an endosymbiont of the shipworm (Distel *et al.*, 2002 ▸). The shipworm harnesses a combination of endogenously produced enzymes, and those produced by *T. turnerae*, to allow it to digest its cellulosic diet as demonstrated in transcriptomic and proteomic analyses (O’Connor *et al.*, 2014 ▸; Sabbadin, Pesante *et al.*, 2018 ▸). *T. turnerae* encodes a single LPMO in its genome, which has been biochemically characterized and found to contain an additional copper site that had not been identified in enzymes from other species (Fowler *et al.*, 2019 ▸). This additional copper binding site was suggested as a potential docking site for a binding partner to deliver electrons to the enzyme. Here, we set out to investigate whether two proteins, TERTU_2913 and TERTU_3803 identified from the *T. turnerae* genome (https://www.genome.jp/kegg/), may represent such an electron-donating binding partner. These proteins were predicted to contain domains that shared sequence similarity with Cbp2D, but with different overall architectures. We have determined X-ray crystal structures for several of these domains as a first step towards elucidating their function and have performed assays, where possible, to assess their potential roles in biomass breakdown. The results that we have obtained appear to be inconsistent with these proteins playing an electron-donating role in LPMO biochemistry, throwing open the question about their redox role in the biology of *T. turnerae*.

## 2. Results and discussion

### 2.1. TERTU_2913 and TERTU_3803 code for redox-domain-containing proteins

**Figure 1:**
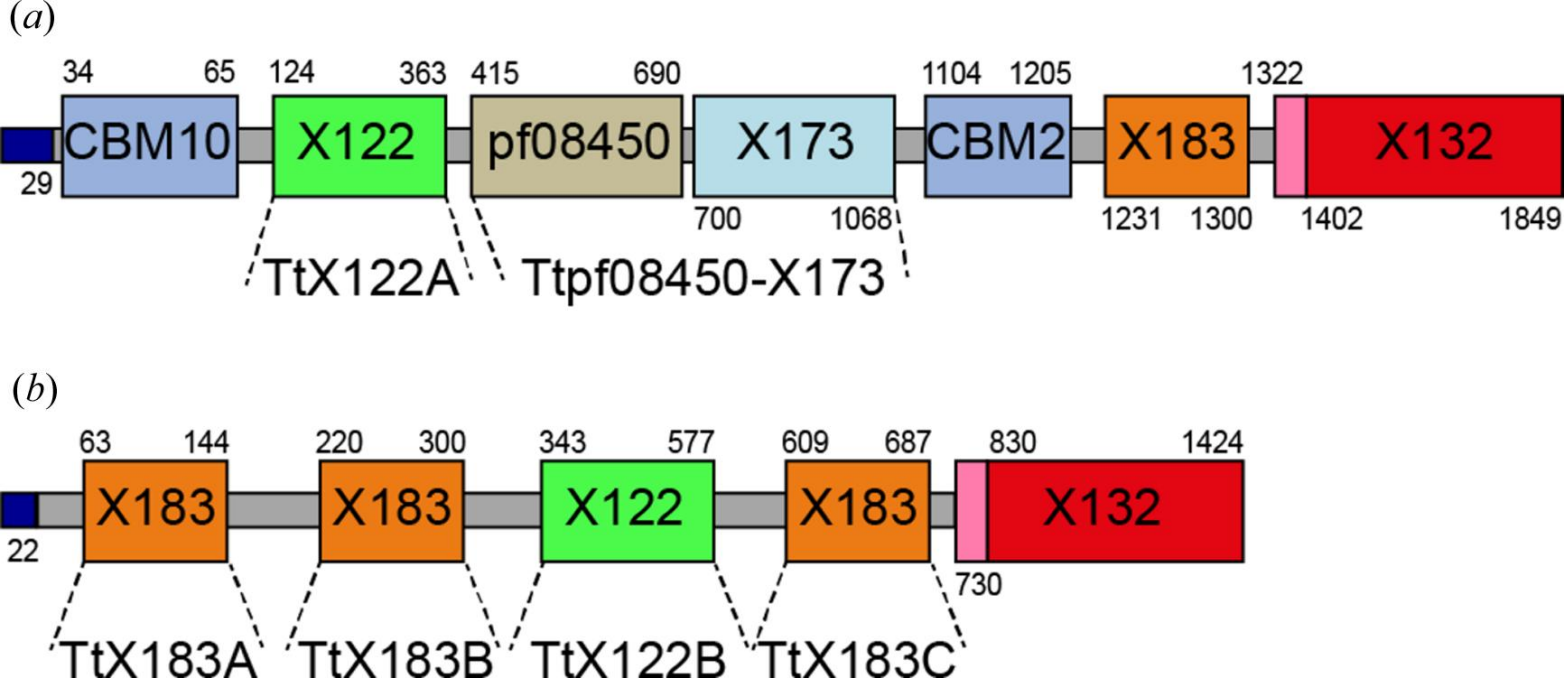
Domain annotation for (*a*) TERTU_3803 and (*b*) TERTU_2913 as used during our analysis. The numbers represent the residues in the linear amino acid sequence where the domain boundaries have been predicted to occur. Flexible linkers are shown in grey and predicted domains are coloured by domain type. The small pink domain at the N-terminal end of the X132 domain in each protein constitutes a DUF1687 domain as annotated in Pfam. The constructs that were expressed and used in the present study are labelled below the full-length proteins. Domains and linkers are not drawn to scale.

The carbohydrate-active enzymes database (CAZy) sets out to classify the domains present within carbohydrate-active enzymes based on sequence, structure and activity (www.cazy.org) (Levasseur *et al.*, 2013 ▸). Whilst annotating the genome of the shipworm symbiont *T. turnerae*, several genes that appeared to code for proteins containing CBMs [see Boraston *et al.* (2004 ▸) and Gilbert *et al.* (2013 ▸) for reviews] with domains of unknown function appended were identified. One of these was TERTU_3803, which contained both a CBM10 and a CBM2 domain attached to a range of distinct modules (via flexible linkers) that did not bare any sequence similarity with catalytic domains typically found within carbohydrate-active enzymes. Such domains of unknown function are defined in CAZy with an X*n* classification (where *n* is a number) until a family member is functionally characterized to allow a more complete definition of the role for that domain. Four domains in TERTU_3803 were therefore designated as X122, X132, X173 and X183 domains, with two other domains annotated with their Pfam (protein family) classifications (Fig. 1 ▸). Using a ‘module walking’ approach (Hemsworth *et al.*, 2014 ▸), we searched for other genes coding for these X-domains. This led to the identification of TERTU_2913, which lacks CBMs but contains predicted X122, X183 and X132 domains (Fig. 1 ▸). These proteins shared some similarity to several proteins that were detected by O’Connor *et al.* (2014 ▸) in their proteomic analysis of the shipworm gut, giving us confidence that these proteins should be produced in the bacterium’s native environment.

Further sequence analysis suggested that X122 domains have distant sequence similarity to cellulose induced protein 1 (Cip1) from *Hypocrea jecorina*, a protein that is upregulated in this organism during growth on cellulose but whose function has not yet been elucidated (Foreman *et al.*, 2003 ▸; Jacobson *et al.*, 2013 ▸). The pf08450-X173 domain pair appeared to have distant sequence similarity to proteins annotated as PQQ-dependent glucose/l-sorbosone de­hydrogenases and sugar lactone lactonases that had not been biochemically characterized, whilst X183 and X132 domains contained CxxCH motifs, which are typically found in *c*-type cytochromes and are also found in *C. japonicus* Cbp2D (Branch *et al.*, 2021 ▸; Gardner *et al.*, 2014 ▸). Signal peptides were identified at the N-terminus of both proteins, suggesting that they are probably secreted, as are LPMOs. Taken together, these sequence analyses suggested that TERTU_3803 and TERTU_2913 could have a role to play in cellulose metabolism, and furthermore that role is likely to be a redox one given the presence of cytochrome domains within their linear amino acid sequences. Following unsuccessful attempts to produce the full-length proteins in *Escherichia coli*, we opted to express individual domains where possible to allow an initial structural and functional investigation into these proteins. We successfully purified the three X183 domains from TERTU_2913, which we term TtX183A, TtX183B and TtX183C; each of the X122 domains from TERTU_3803 and TERTU_2913, which will henceforth be referred to as TtX122A and TtX122B, respectively; and the pf08450-X173 domain pair from TERTU_3803 for further characterization (Fig. 1 ▸).

### 2.2. Structures of TtX183A and TtX183B reveal *c*-type-like cytochrome domains

**Figure 2:**
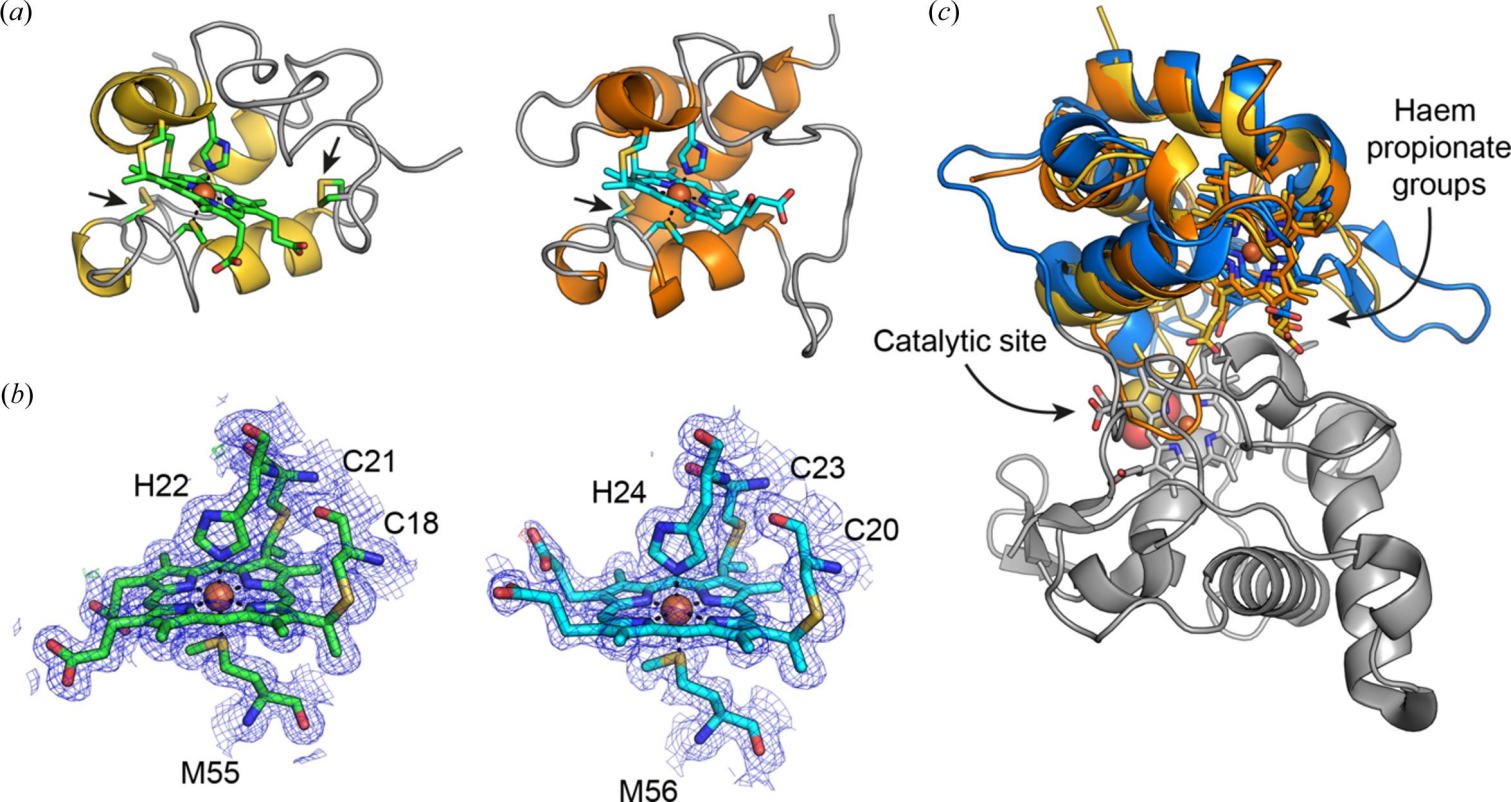
(*a*) Cartoon representations of the TtX183A (left) and TtX183B (right) domain structures. The haem cofactor is shown in stick form, coloured by atom type, with carbons shown in green or cyan. The cysteines that covalently link to the haem moiety are also shown as sticks together with the axial me­thio­nine and histidine that coordinate the central iron atom. The positions of cysteines that form disulfide bonds are also shown as sticks and are highlighted by arrows. (*b*) Representative electron-density maps for TtX183A (left, with green carbon atoms) and TtX183B (right, with cyan carbon atoms). The 2*F*_obs_ − *F*_calc_ map for each structure is shown in blue contoured at 1σ, and the *F*_obs_ − *F*_calc_ map shows positive density in green and negative density in red contoured at 4σ. (*c*) The superposition of TtX183A (yellow) and TtX183B (orange) with SoxA (PDB ID 4v2k) in which the catalytic domain has been coloured grey and the electron-transfer domain has been coloured blue. The superpositions demonstrate how the haem propionates may be buried within a larger protein to facilitate electron transfer to or from a catalytic site.

TtX183A, TtX183B and TtX183C, which all derive from TERTU_2913, were expressed and purified from the *E. coli* periplasm following co-expression with the cytochrome maturation machinery encoded on the pEC86 plasmid (Arslan *et al.*, 1998 ▸). All three proteins underwent crystal trials but only TtX183A and TtX183B formed diffracting crystals. The structure for TtX183A was determined via single-wavelength anomalous dispersion from the anomalous signal generated by the haem iron to a resolution of 1.4 Å (Table S1 of the supporting information). The resulting model was subsequently used to determine the structure of TtX183B via molecular replacement to 1.8 Å resolution (Table S1). The polypeptide chain in each of TtX183A and TtX183B fold around the haem to produce a tertiary structure dominated by α-helices, as expected for small *c*-type cytochromes [Fig. 2 ▸(*a*)]. Both proteins show a covalently bound haem molecule that is attached to the polypeptide via two Cys residues at positions 18 and 21 in TtX183A and positions 20 and 23 in TtX183B. These represent the two cysteines present in the conserved CxxCH pentapeptide motif typical of *c*-type cytochromes. The haem iron coordination sphere is completed in both structures with histidine (His22 in TtX183A and His24 in TtX183B) and me­thio­nine (Met55 and Met56 in TtX183A and TtX183B, respectively) sidechains in the axial positions around the central iron of the haem molecule [Fig. 2 ▸(*b*)]. Additionally, TtX183A and TtX183B are each stabilized by disulfide bonds [Fig. 2 ▸(*a*)]. There have been suggestions in the past that disulfides could play functional roles in electron-transfer proteins such as cytochrome *c*_6a_ (Howe *et al.*, 2006 ▸; Schlarb-Ridley *et al.*, 2006 ▸). Subsequent work, however, showed they were more than likely present for structural stability (Mason *et al.*, 2012 ▸), and so given the extracellular nature of the proteins under study here, we consider the disulfides are more than likely present for stability in TtX183A and TtX183B as well.

To examine the structural similarities within our TtX183 domain structures, we compared TtX183A with TtX183B and found that the structures superposed with one another with an r.m.s.d. of 1.12 Å over 59 Cα positions. We also superposed the structures with CjX183, the X183 domain from *C. japonicus* Cbp2D that we recently analysed (Branch *et al.*, 2021 ▸), which overlaid with r.m.s.d.s of 1.49 Å over 62 Cαs and 0.92 Å over 77 Cαs for TtX183A and TtX183B, respectively. These superpositions reveal that the haem moiety is relatively solvent exposed in all of the X183 structures; however, the loop connecting helix 2 with helix 3 in CjX183 is significantly shorter in both of the TtX183 domains (Fig. S1 of the supporting information). In CjX183 this loop interacts with the haem propionate groups, meaning that these groups are considerably more solvent exposed in TtX183A and TtX183B, which probably has knock-on effects on the stability of the reduced state in these domains (discussed later). In addition, this loop varies most between TtX183A and TtX183B in our superpositions [Figs. S1(*a*) and S1(*b*)], which is likely to have significant effects on the redox properties of each domain when compared with one another.

A broader comparison of TtX183A and TtX183B against other structures in the Protein Data Bank (PDB) using the *Dali* server (Holm, 2020 ▸) revealed that these domains shared highest structural similarity with the *c*-type cytochrome domain present in the thio­sulfate de­hydrogenase SoxA (PDB ID 4v2k; Grabarczyk *et al.*, 2015 ▸), superposing with r.m.s.d.s of 2.3 and 2.1 Å over 73 and 70 amino acids, respectively. This is a similar result to that obtained when we compared CjX183 against the PDB previously (Branch *et al.*, 2021 ▸). SoxA and related proteins function in sulfate metabolism, and the domain to which the X183s are a close structural match is present to shuttle electrons away to a terminal electron acceptor following catalysis at a second haem molecule in a separate catalytic domain [Fig. 2 ▸(*c*)] (Grabarczyk *et al.*, 2015 ▸). As such, the haem propionate groups are buried close to the catalytic haem in SoxA to allow efficient electron transfer from the active site, whereas the equivalent groups are solvent exposed in our structures [Fig. 2 ▸(*c*)]. Since the X183 domains under study here are part of a larger protein, it is possible that these domains are also present within TERTU_2913 to mediate the transfer of electrons to, or from, a catalytic domain within the protein, or to an enzyme partner protein.

In a bid to gain some insight into the overall architecture of full-length TERTU_2913, we used *AlphaFold*2 (Jumper *et al.*, 2021 ▸) to predict its structure. Five models were generated in which the structures of the individual domains were consistent with one another across all models and were predicted with high confidence by the program [Fig. S2(*a*)]. Interestingly, the long flexible linkers in the protein should have provided *AlphaFold*2 with relative freedom to place the domains freely in space, but in all models the X183 domains were placed near to one another [Fig. S2(*b*)]. The X183A and X183B structures that we determined experimentally could also be superposed onto these models with low r.m.s.d. values allowing us to visualize the possible positioning of the haem moieties within these domains [Fig. S2(*c*)]. The *AlphaFold*2 models also provide a first insight into the probable structure of the X132 domain at the C-terminus of the protein, for which there are no structurally or functionally characterized homologues to our knowledge. The X132 domain contains an additional CxxCH motif that would be expected to be haem bound, and in the *AlphaFold*2 models this motif is routinely placed close to the X183 domains as well [Fig. S2(*c*)]. Whilst the protein is clearly going to have significant flexibility and these domains are likely to be mobile, this analysis suggests a potential co-localization of the haem moieties in the native protein and supports the hypothesis that the X183 domains are likely to function in electron transfer to or from a yet-to-be-identified electron acceptor or donor. We therefore set out to test our initial hypothesis that TERTU_2913 and TERTU_3803 may function in LPMO biochemistry by testing the reduced X183 domains that we had purified as electron donors to an LPMO.

### 2.3. The TtX183s do not donate electrons to TtAA10 to attack cellulose

We tested all three of the isolated TtX183 domains as electron donors to TtAA10, the cognate LPMO from *T. turnerae* (Branch *et al.*, 2021 ▸; Fowler *et al.*, 2019 ▸). We first prepared the reduced X183s by the addition of an excess of ascorbate, which was subsequently removed using PD-10 desalting columns. These samples were immediately used in place of small-molecule reducing agents in LPMO activity measurements on cellulose by incubation with phospho­ric acid swollen cellulose (PASC). Matrix-assisted laser desorption ionization time of flight (MALDI-TOF) mass spectrometry was then used to detect oxidized products liberated by LPMO action. Positive controls using ascorbate as the electron source recapitulated the results seen previously in which oxidized oligosaccharide products were liberated from the substrate by the LPMO (Fig. S3) (Fowler *et al.*, 2019 ▸). When the reduced X183s were used as the electron source, however, we were unable to detect any oxidized products being released. This result contrasted with our previous observations using CjX183, so we further probed the redox properties of these domains in search of an explanation for the apparent lack of electron donation to the LPMO.

### 2.4. TtX183 domains display redox properties in line with serving an electron-transfer function but lose their electrons rapidly

**Figure 3:**
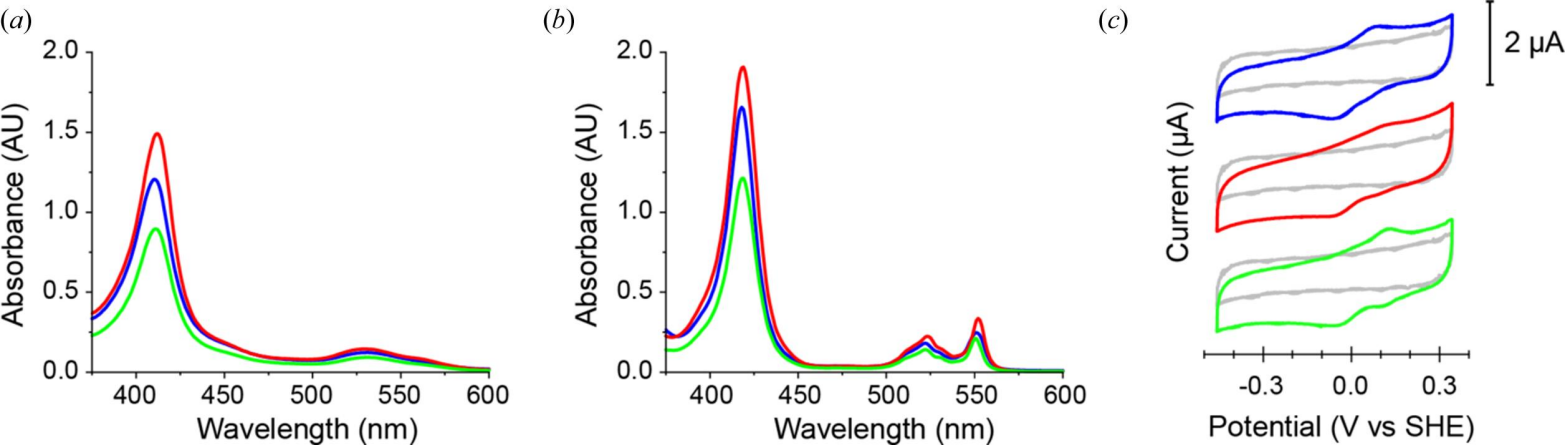
Typical UV–Vis spectra of (*a*) ferric (oxidized) and (*b*) ferrous (reduced) forms of the TtX183 domains. Spectra for TtX183A, TtX183B and TtX183C are shown in blue, red and green, respectively. The α- and β-bands display maxima at 523 and 552 nm in the ferrous state, which flatten out to a broad peak when oxidized. The Soret band also shifts from 419 to 409 nm upon oxidation. All three spectra overlay very closely demonstrating that each X183 domain possesses similar haem environments and electronic characteristics. (*c*) Characteristic CVs at pH 7.0 for protein films of TtX183A, TtX183B and TtX183C on a pyrolytic graphite-edge working electrode coloured as in (*a*) and (*b*). Blank measurements for the pyrolytic graphite-edge working electrode in the absence of protein film are shown in grey. The midpoint potentials of the redox couples observed in the CVs are used to determine the reduction potentials of the haem iron in each domain.

We first analysed the spectroscopic properties of TtX183A, TtX183B and TtX183C using UV–Visible light spectroscopy. As would be expected for a *c*-type cytochrome, the visible spectra for these domains were all dominated by strong absorbance from the Soret band at 409 nm in the ferric state, and a rise in absorbance by the α and β bands at 550 and 520 nm, respectively, following reduction to the ferrous state [Figs. 3 ▸(*a*) and 3 ▸(*c*)]. In order to obtain a spectrum of the ferrous state, it was necessary to either maintain an excess of ascorbate in the sample or prepare the sample in an anaerobic chamber and then seal it prior to UV–Vis measurements being taken. Samples prepared in an aerobic environment were found to oxidize rapidly before a spectrum of the fully reduced state could be measured. This demonstrates that the ferrous state in these X183 domains is not very stable; hence, during our attempts to measure LPMO activity, rather than donating electrons to the enzyme, the electrons were more likely lost to O_2_.

To clarify further whether the observed loss of electrons was a result of the redox properties of the haem, we measured the reduction potentials of each domain using cyclic voltammetry. The proteins were individually dispensed on a carbon electrode and allowed to form a film in an anaerobic environment. The electrode was then submerged in buffer and cyclic voltammograms (CVs) were recorded. The resulting midpoint reduction potentials for TtX183A, TtX183B and TtX183C were thus determined to be +11, +119 and +124 mV versus the standard hydrogen electrode (SHE), respectively [Fig. 3 ▸(*c*)]. *c*-type cytochromes have been identified with midpoint reduction potentials ranging from −390 mV to +450 mV versus the SHE; however, those with His/Met ligation, as observed here for the X183 domains, would typically be expected to have midpoint reduction potentials at the higher end of this range (Liu *et al.*, 2014 ▸). The reduction potentials we have measured for these domains are thus slightly lower than expected, but are also all lower than those that have been typically reported for LPMOs (Aachmann *et al.*, 2012 ▸; Borisova *et al.*, 2015 ▸; Forsberg *et al.*, 2014 ▸; Hemsworth *et al.*, 2013 ▸; Zouraris *et al.*, 2018 ▸). One would, therefore, expect that these domains should be capable of electron donation to these enzymes. As described earlier, in the structures of TtX183A and TtX183B, the haem propionate groups are highly solvent exposed where in CjX183 a loop was found to interact with these groups (Fig. S1). This is likely to provide an easier exit route for electrons when O_2_ is present, explaining the apparent lack of stability of the reduced state for the TtX183s compared with CjX183 (Branch *et al.*, 2021 ▸).

The rapid oxidation of the TtX183 domains that we observed was unexpected and explains the lack of electron donation to TtAA10. Without the full-length TERTU_2913 protein for biochemical characterization it is hard to say whether these results are functionally relevant. Most extracellular multi-domain *c*-type cytochrome containing proteins carefully position their multiple haem molecules to allow electron transfer across long distances and often contain catalytic domains (Edwards *et al.*, 2020 ▸). To try to provide additional insight into potential functions for TERTU_2913 and TERTU_3803, we therefore set out to further characterize some of the non-haem-containing domains from these proteins.

### 2.5. Ttpf08450-X173 does not bind PQQ nor does it have glucose de­hydrogenase activity

*BLAST* searches using the Ttpf08450-X173 sequence returned closest sequence matches to proteins that had been annotated as PQQ-dependent d-glucose/l-sorbosone de­hydrogenases. PQQ-dependent enzymes, annotated as AA12s in CAZy, have been demonstrated to act as effective LPMO activators and may have an important role to play in biomass breakdown (Várnai *et al.*, 2018 ▸). We therefore produced Ttpf08450-X173 for structure-function studies from a pET26b construct, which directed the protein to the *E. coli* periplasm and allowed easy purification of the protein via a C-terminal histidine tag. The protein could be readily concentrated to 10–20 mg ml^−1^, but following several rounds of crystal trials, we were unable to obtain crystals of this di-domain construct to allow its structural analysis. We therefore pursued assays to try to establish a potential role for these domains in the absence of structural information. PQQ-dependent d-glucose/l-sorbosone de­hydrogenases should oxidize d-glucose to d-glucono-1,5-δ-lactone using the PQQ cofactor, which is reduced to its quinol form. We initially attempted to assay this activity for Ttpf08450-X173 as purified using the d-gluconic acid/d-glucono-δ-lactone assay kit from Megazyme, but we were unable to detect any activity following incubation with glucose. Mass spectrometry and UV–Vis spectroscopy subsequently suggested that PQQ had not been co-purified with the protein. Performing the assay with the addition of PQQ still failed to yield any sign of enzyme activity in assays, so we attempted to detect PQQ binding to the protein using isothermal titration calorimetry (ITC). Experiments were performed in the presence and absence of Ca^2+^, which is essential for PQQ binding in many PQQ-dependent enzymes (Oubrie *et al.*, 1999 ▸; Stines-Chaumeil *et al.*, 2020 ▸), but we were unable to detect PQQ binding under any of the conditions that we tested. This suggests that these domains cannot bind to PQQ and so may not provide a PQQ-dependent de­hydrogenase activity after all. It is possible that these domains may bind to an alternative redox cofactor, but we were unable to find examples in the literature of cofactors other than PQQ binding to such proteins. In the absence of structural information and given the diversity of quinones that can exist in nature, we did not probe further for other redox cofactors that may be utilized by these domains.

### 2.6. TtX122A and TtX122B have β-jelly-roll folds like some glycoside hydro­lases, polysaccharide lyases and lectins

In addition to our work on Ttpf08450-X173, we produced TtX122A and TtX122B, representing the X122 domains from TERTU_3803 and TERTU_2913, respectively. These were produced as individual proteins in the periplasm of *E. coli*, and were purified for structural and biochemical studies via a C-terminal histidine tag. Both domains readily crystallized, which allowed the structure of TtX122A to be determined first to 1.8 Å via multiple-wavelength anomalous dispersion using protein that had seleno­methio­nine (Se-Met) incorporated during protein expression (Table S2). This subsequently allowed a structure of the native protein to be determined to 1.5 Å resolution. The electron density was readily interpretable allowing two molecules of TtX122A to be modelled in the asymmetric unit without breaks in the chain representing residues 3 to 244 of the protein coded for in our construct. With the structure of TtX122A in hand, the structure of TtX122B could be solved to 2.2 Å via molecular replacement using a single protomer of TtX122A as an initial search model (Table S2). Three molecules of TtX122B were present in the asymmetric unit, with chain B’s electron density most readily traced allowing modelling of residues 2 to 235 without breaks in the chain. Chains A and C had some areas that displayed more disorder, so there were breaks in the model between residues 190 and 192 in chain A and residues 162 and 165 in chain C. In addition to protein and water molecules, two magnesium ions were modelled into the electron density on each protomer in TtX122A based on coordination geometry and bond lengths [Fig. S4(*a*)]; these ions presumably bound from the crystallization medium. A single calcium ion could also be modelled in each protomer of both TtX122A and TtX122B [Fig. S4(*b*)]; each located in a site with coordination predominantly mediated by main-chain carbonyl groups that has been implicated in stabilizing the structure in other proteins with this fold (Jacobson *et al.*, 2013 ▸). The protein had not been exposed to Ca^2+^ since purification, so these ions must have co-purified with the protein from the cells.

**Figure 4:**
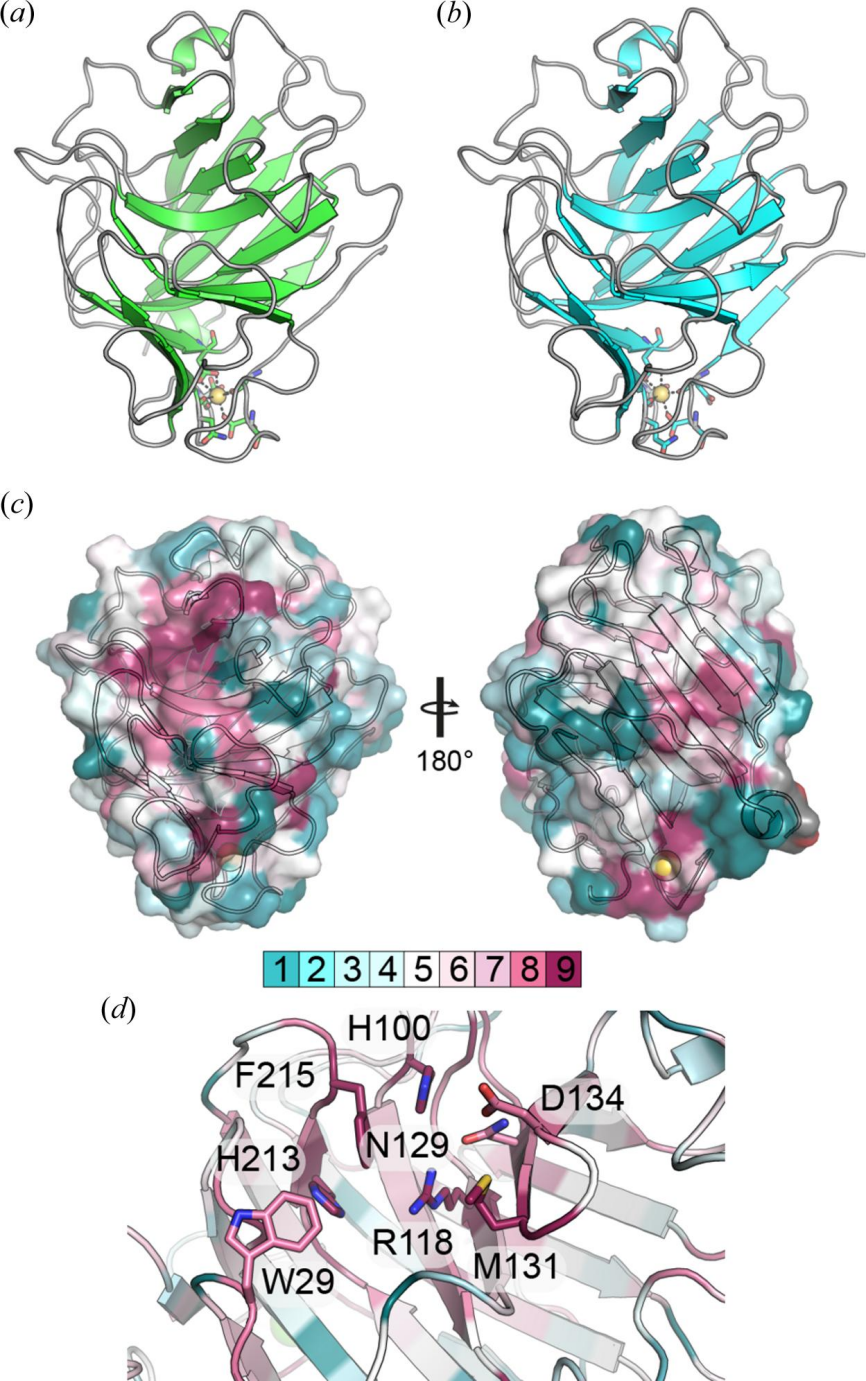
The overall fold of (*a*) TtX122A and (*b*) TtX122B shown at the same viewing angle. In both cases, the calcium binding site is represented with a yellow sphere with coordinating residues shown as sticks. (*c*) The molecular surface of TtX122A shown at two viewing angles rotated by 180° and coloured by sequence conservation resulting from *ConSurf* analysis using 648 sequences. On the left, the most highly conserved surface is found surrounding Arg118 and is in the vicinity of active or ligand binding sites in other proteins with this fold. On the right, the most conserved region surrounds the calcium ion shown in yellow. (*d*) A close-up view of the conserved amino acids centred around Arg118, which may represent a conserved active or ligand binding site within the X122 family. The most highly conserved residues in this region are shown as sticks and coloured by atom type, with carbon atoms coloured according to the *ConSurf* score as shown in (*c*).

To check the oligomeric state of our TtX122 structures, *PISA* analysis was performed, which revealed that there were no significant molecular interfaces between protomers in the crystal for either structure, suggesting that the molecules would be expected to be monomeric in solution (Krissinel & Henrick, 2007 ▸). Our subsequent structural analysis therefore focused on single protomers from each structure. The overall structures of TtX122A and TtX122B reveal that these domains adopt a β-jelly-roll fold [Figs. 4 ▸(*a*) and 4 ▸(*b*)]. The two structures are highly similar and can be superposed with one another with an r.m.s.d. of 0.42 Å over 228 Cα positions [Fig. S3(*a*)]. Since the structures are so similar, the remainder of our analysis will focus on TtX122A, which represents the highest resolution and hence the best resolved structure of these two domains. Structural comparisons using the *Dali* server revealed close structural matches between TtX122A and Cip1 from *H. jecorina* [PDB ID 3zyp, r.m.s.d. = 2.7 Å over 201 Cαs (Jacobson *et al.*, 2013 ▸)], a PL20 glucuronan lyase from *Trichoderma reesei* called TrGL [PDB ID 2zzj, r.m.s.d. = 3.0 Å over 179 Cαs (Konno *et al.*, 2009 ▸)] and the lectin domain from mouse galactocerebrosidase [PDB ID 3zr5, r.m.s.d. = 2.7 Å across 166 Cαs (Deane *et al.*, 2011 ▸)] [Figs. S3(*b*)–S3(*d*)]. These matches share only 21, 12 and 11% sequence identity, respectively, with TtX122A, demonstrating that the X122 domains under study here have significantly divergent sequences relative to previously characterized examples of proteins containing similarly structured domains. In addition, these structural matches demonstrate the diversity of roles to which the β-jelly-roll fold has been applied throughout evolution (Viborg *et al.*, 2019 ▸). Cip1, which represents the closest structural and sequence match to TtX122A, is upregulated in *H. jecorina* during growth on cellulose; however, a biochemical function for this protein has not yet been determined (Jacobson *et al.*, 2013 ▸). PL20s are catalytic domains in their own right and catalyse the cleavage of glycosidic linkages in glucuronan via β-elimination (Konno *et al.*, 2009 ▸), whilst the lectin domain from galactocerebrosidase is important in constructing the enzyme’s active site and mediates interactions with saposin-A, which delivers the substrate to the enzyme (Deane *et al.*, 2011 ▸; Hill *et al.*, 2018 ▸),

Since there was limited functional similarity between the proteins identified in our structural comparisons, we performed a sequence alignment of 648 X122 family members and mapped the sequence conservation of each amino acid onto the surface of TtX122A using the *ConSurf* server (Ashkenazy *et al.*, 2016 ▸). This revealed two significantly conserved patches on the protein surface [Fig. 4 ▸(*c*)], one of which was located around the calcium binding site implicated in stabilizing the fold, whilst the other forms a conserved surface on the inner face of the β-jelly-roll fold centred around Arg118 [Fig. 4 ▸(*d*)]. Closer examination of this conserved patch shows that the most highly conserved residues are represented by Arg118, His100, Phe215, His213 and Met131, together with the slightly less conserved Trp29, Asn129 and Asp134 residues [Fig. 4 ▸(*d*)]. These residues reside on the inner face of the β-jelly-roll fold, which is often home to an active site or ligand binding site in many domains with this fold. The analysis by Jacobson *et al.* (2013) of Cip1 highlighted a similar set of residues as potentially being the active site if the domain was a polysaccharide lyase. Indeed, Arg100 in Cip1 (equivalent to Arg118 in TtX122A) was implicated as a potential catalytic residue, as arginine can act as a base to form a salt bridge with the carboxyl­ate present in the acidic substrates of lyases (Jacobson *et al.*, 2013 ▸; Konno *et al.*, 2009 ▸). However, Cip1 was not demonstrated to have any significant lyase activity (Jacobson *et al.*, 2013 ▸).

To take the analysis further, we provided the coordinates of the cluster of seven residues that were identified from the *ConSurf* analysis to *ASSAM* (Nadzirin *et al.*, 2012 ▸). *ASSAM* is a tool that can be used to search for similar arrangements of amino acids in other proteins, irrespective of the overall fold of the protein. This can therefore be useful for identifying functional sites from other proteins that may not be evolutionarily related to the protein of interest. The *ASSAM* search returned 1073 PDB right-handed superpositions in which between three and five residues matched with subsets of the residues provided from TtX122A, with r.m.s.d.s ranging from 0.85 to 1.80 Å. Whilst many of these sites did not appear to be active sites or binding sites, examining structures that contained heteroatoms (that were not metal ions or likely solute molecules) within close proximity of the matching residues revealed several structures that contained carbohydrate moieties within close proximity. These hits included proteins annotated as β-glucosidase, α-amylase, phospho­glycerate mutase, α-1,4-glucan lyase, fucose binding lectin and fructose binding protein (Fig. S6). Close examination of the carbohydrate placement in the superpositions with the full structure of TtX122A suggested that binding of similar carbohydrates to those identified in this screen was unlikely given clashes with other regions of the protein. This made it challenging to identify potential substrates for enzyme/binding assays but lends some support to the notion that these domains may be involved in binding to or have activity on a carbohydrate substrate in some capacity.

### 2.7. TtX122 domains do not show clear enzymatic activity or carbohydrate binding function

Since our structural analysis of TtX122A and TtX122B provided few clues towards the function that these domains could perform in these proteins, we set out to investigate whether they could harbour some catalytic activity on polysaccharides found in nature. Given the presence of CBM10 and CBM2 modules in the amino acid sequence of TERTU_3803, we conducted some initial enzyme screens using thin-layer chromatography (TLC) to probe for potential activity on a range of polysaccharides including cellulose and chitin. This initial analysis did not reveal any activity on the substrates tested and was too low throughput to allow wide-range screening. We therefore attempted to screen for potential activity on a broader range of substrates using microarrays in the epitope depletion method described by Vidal-Melgosa *et al.* (2015 ▸). Briefly, this method relies on enzyme activity disrupting epitopes recognized by monoclonal antibodies (mAbs) that are specific to select polysaccharides spotted out in the microarray. Abrogation of antibody binding can therefore be used to unveil enzyme activity and the substrate preferences of carbohydrate-active enzymes. TtX122A and TtX122B were screened against a range of soluble polysaccharides from terrestrial and marine sources using this approach (Table S3 and Fig. S7). The microarrays were probed using mAbs and CBMs specific to the polysaccharides of interest and then a secondary alkaline-phophatase conjugated antibody was used to develop colour, the intensity of which reflects the level of primary antibody binding. The resulting arrays were scanned and the mean spot intensity for each sample was measured. Heat plots were then generated by comparing the intensity measurements for X122 treated samples against a no-enzyme control array, revealing the fold change in antibody binding observed in each case. Very similar results were observed for both TtX122A and TtX122B (Fig. S7), in which there were only very small changes in antibody binding following X122 treatment in the case of some alginates (predominantly PAA and PAU, see Table S3) and wheat arabinoxylan, where twofold or threefold changes in antibody binding could be observed. When compared with the positive controls, these results appeared insignificant, as up to a 19-fold loss of antibody binding could be observed when polygalacturonic acid hydro­lase and a pectate lyase were used as positive controls.

Without a clear-cut activity for the X122 domains that we had isolated, we considered whether these domains may instead represent novel carbohydrate binding domains given their structural similarity to lectin-like domains. We therefore produced and purified new constructs for both TtX122A and TtX122B in which green fluorescent protein (GFP) had been fused to the protein via a short linker at either the N- or C-terminus. We also included a construct in which the CBM10 N-terminal to TtX122A from TERTU_3803 was included in the construct to be used alongside the well characterized CBM2b1-2:GFP binding domain as a positive control (Hervé *et al.*, 2010 ▸). Tobacco-stem cross sections were incubated with these GFP-fusion proteins and subsequently examined using fluorescence microscopy to determine whether the domains had significant affinity towards plant cell-wall polysaccharides present in these tissues. The positive control gave a clear fluorescent signal under the microscope, as observed previously (Hervé *et al.*, 2010 ▸) (Fig. S8). We were unable to detect any fluorescence in the tissue from the X122A:GFP or X122B:GFP fusion protein alone and only a weak signal was observed for the CBM10-X122A:GFP protein (Fig. S8). We therefore conclude that these domains do not mediate binding to plant cell-wall polysaccharides to a significant degree.

## 3. Conclusions

The discovery of LPMOs has generated considerable renewed interest in redox processes as related to polysaccharide degradation and utilization in the environment (see Beeson *et al.* (2015 ▸), Ciano *et al.* (2018 ▸), Hemsworth *et al.* (2015 ▸) and Vaaje-Kolstad *et al.* (2017 ▸) for several reviews). In fungi, LPMOs operate in the presence of a diversity of other secreted redox proteins, with the interplay between these proteins being an active area of research (Garajova *et al.*, 2016 ▸; Kracher *et al.*, 2016 ▸; Phillips *et al.*, 2011 ▸; Sygmund *et al.*, 2012 ▸; Várnai *et al.*, 2018 ▸). For bacteria, the redox-protein environment for LPMOs is not well understood. We therefore elected to study TERTU_2913 and TERTU_3803, which represent secreted proteins from *T. turnerae* that may have a role to play in this context. We have demonstrated that the X183 domains from these proteins are *c*-type cytochromes with probable electron-transfer functions; however, under the conditions we tested, these domains were not capable of delivering electrons to LPMOs for activity, and they showed considerable instability of the reduced state in the absence of an exogenous reducing agent and in the presence of O_2_. Our structures of two X122 domains revealed that they adopt a β-jelly-roll fold, and our sequence and structural analysis highlighted a patch of conserved residues that may represent a binding or catalytic site for a substrate. We were unable to identify a potential substrate for this domain, nor were we able to detect activity or cofactor binding for the pf08450-X173 domain fusion that was predicted to be a PQQ-dependent de­hydrogenase. These results highlight the challenges of working with such multi-modular proteins that are active in a redox setting. Where catalytic domains for glycoside hydro­lases and other enzymes can often be characterized in isolation, any catalytic activities present within TERTU_2913 and TERTU_3803 may be dependent upon the other domains found within the linear polypeptide, or other redox partners present in the environment, for their activity. The X132 domains present at the C-terminus of TERTU_3803, TERTU_2913 and *C. japonicus* Cbp2D also contain a CxxCH motif typical of *c*-type cytochromes with a novel sequence providing further intrigue as to their function. Our analysis provides a first glimpse into these multi-haem *c*-type cytochromes that have not been studied previously, to our knowledge. *T. turnerae* resides in the gills of the shipworm, and many of the proteins that it secretes, including some like those studied here, are known to reach the caecum where cellulose degradation takes place (Distel *et al.*, 2002 ▸; Sabbadin, Pesante *et al.*, 2018 ▸). Large multi-haem-containing *c*-type cytochromes are often harnessed by some bacteria to deliver electrons across long distances to terminal electron acceptors (Edwards *et al.*, 2020 ▸). TERTU_2913 and TERTU_3803 may play such a role in the symbiotic relationship between these bacteria and the shipworm. We hope that our structural and functional analysis will lay the groundwork for further characterization of these, and related proteins, which has the potential to unveil previously unseen roles for extracellular *c*-type cytochromes in bacteria.

## 4. Materials and methods

### 4.1. Expression and purification of TtX183A, TtX183B and TtX183C

The coding sequence for TERTU_2913 (GenBank: ACR10928.1) was identified in the *T. turnerae* T7901 genome sequence (NCBI nucleotide CP001614) and was synthesized with codon optimization for expression in *E. coli* by Genewiz. The regions coding for TtX183A (nucleotides 184 to 438), TtX183B (nucleotides 649 to 891) and TtX183C (nucleotides 1840 to 2082) were subsequently cloned into the pCW-LIC^Amp^ vector (a gift from Cheryl Arrowsmith, Addgene plasmid 26098) downstream of a pelB leader sequence using polymerase incomplete primer extension (PIPE) cloning (Klock & Lesley, 2009 ▸). Proteins were subsequently produced in BL21(DE3) cells (Invitrogen) by the co-expression of genes for haem maturation from the pEC86^Cam^ vector (Arslan *et al.*, 1998 ▸). Then, 1 l cultures of cells were grown in 2xYT media (16 gl^−1^ tryptone, 10 gl^−1^ yeast extract, 6 gl^−1^ NaCl) at 37°C in baffled flasks shaking at 180 r min^−1^ until an A_600_ of 0.6 was reached. The temperature was then lowered to 16°C and gene expression was induced by the addition of IPTG to a final concentration of 1 m*M*. Cultures were allowed to grow overnight before cells were harvested by centrifugation at 5000*g* for 20 min.

Bacterial pellets were resuspended in 3x volumes of 20 m*M* Tris pH 8.0, 150 m*M* NaCl and 20% sucrose before 40 µl of 10 mg ml^−1^ lysozyme was added for every gram of cell paste. The cells were incubated in ice for 1 h with occasional agitation before 60 µl of 1 *M* MgSO_4_ was also added to the sample. Following a further 20 min on ice, the suspension was centrifuged at 12 000*g* for 20 min. The supernatant containing periplasmic fraction 1 was removed and the pellet was resuspended in 3x volumes of ice-cold Milli-Q water. This was incubated on ice for 1 h before being centrifuged again at 12 000*g* for 20 min. The supernatant was removed and combined with periplasmic fraction 1 forming the final periplasmic lysis fraction. Proteins were subsequently purified from here by application to a 5 ml HisTrap FF column (Cytiva) that had been equilibrated in Buffer A (20 m*M* Tris–HCl pH 8.0, 150 m*M* NaCl and 30 m*M* imidazole). The protein was then eluted using a linear gradient to a final concentration of 300 m*M* imidazole over 20 CVs collecting 1.8 ml fractions. The fractions were analysed by SDS–PAGE, with those containing the desired protein pooled, concentrated using a 3 kDa cut-off Vivaspin concentrator (Sartorius) to less than 1 ml volume and applied to a 16/600 Superdex 75 column (Cytiva) that had been equilibrated in 20 m*M* Tris pH 8.0, 150 m*M* NaCl. Again, the fractionated samples were analysed by SDS–PAGE, and selected fractions were pooled and concentrated using a 3 kDa molecular-weight cut-off concentrator. Sample concentrations were determined by measurement of the A_410_ for the haem cofactor and an extinction coefficient of 106 000 *M* ^−1^cm^−1^.

### 4.2. Crystallization, X-ray data collection and structure determination for TtX183A and TtX183B

Crystals for TtX183A were obtained using the hanging-drop vapour diffusion method, with the reservoir containing 20–25%(*w*/*v*) PEG 6000, 1 *M* LiCl_2_ and 0.1 *M* citrate pH 4.0, whilst the condition for forming TtX183B contained 15–25%(*w*/*v*) PEG 6000, 200 m*M* sodium acetate pH 5.0 and 200 m*M* NaCl. In both cases, 1:1 µl drops were set up by mixing TtX183A at 5 mg ml^−1^ and TtX183B at 10 mg ml^−1^ with mother liquor, and screens were incubated at 20°C for 1 week to allow crystals to grow.

For data collection, single crystals were transferred to a cryoprotectant solution containing the reservoir solution and 15% glycerol, before flash cooling in liquid nitro­gen. X-ray diffraction data were measured for TtX183A on the MASIF-1 automated data-collection beamline at the European Synchrotron Radiation Facility, whilst data were collected for TtX183B on beamline I24 at Diamond Light Source. The diffraction data in both cases were processed using *XDS* (Kabsch, 2010 ▸) and *CCP*4*i*2 (Potterton *et al.*, 2018 ▸). The structure for TtX183A was determined using single-wavelength anomalous dispersion using the anomalous signal generated by the haem iron in the *SHELXC/D/E* pipeline (Sheldrick, 2008 ▸, 2015 ▸). The structure for TtX183B was determined by molecular replacement using the model of TtX183A with haem and water molecules removed as the search model. All model building and refinement was performed in *Coot* (Emsley *et al.*, 2010 ▸) and *REFMAC*5 (Murshudov *et al.*, 1997 ▸), and the final models were validated using *MolProbity* (Williams *et al.*, 2018 ▸).

### 4.3. LPMO activity assays using matrix-assisted laser desorption ionization mass spectrometry (MALDI-MS) and TtX183s as electron source

TtAA10 activity assays were set up using PASC [prepared according to Wood (1988 ▸)] or Avicel as the main substrate. First, 1 ml samples were prepared in 50 m*M* sodium acetate pH 6.0 buffer containing 1 mg ml^−1^ Avicel or PASC and 1 µ*M* TtAA10. Then, 1 m*M* ascorbate was used as the electron donor in positive controls. Where TtX183A, TtX183B or TtX183C were used as the electron source, the haem was chemically reduced first by the addition of 1 m*M* ascorbate, which was subsequently removed by passing the protein down a PD-10 desalting column before the protein was added to assays at 100 µ*M*. Reactions were incubated rotating end-over-end on a tube rotator (Stuart Scientific) overnight at room temperature. Prior to mass-spectrometric analysis, the samples were centrifuged at 10 000*g* for 1 min to pellet any solid material.

For MALDI-MS measurements, 1 µl of sample was mixed with an equivalent volume of 10 mg ml^−1^ 2,5-di­hydroxy­benzoic acid in 50% aceto­nitrile, 0.1% tri­fluoro­acetic acid on a Bruker SCOUT-MTP 384 target plate. The spotted samples were then dried in air under a lamp before being analysed by mass spectrometry on an Ultraflex III MALDI-TOF/TOF instrument (Bruker), as described by Hemsworth *et al.* (2014 ▸).

### 4.4. UV–Vis analysis of TtX183A, TtX183B and TtX183C

UV–Vis absorption spectra for TtX183A, TtX183B and TtX183C were measured in a 1 cm quartz cuvette on a Cary60 spectrophotometer (Agilent). To prepare oxidized X183 domains, 1 m*M* potassium ferricyanide was added to 100 µ*M* protein, which was then passed down a PD-10 desalting column to remove excess oxidizing agent prior to measurements being taken. To prepare reduced X183 proteins, 1 m*M* ascorbate was added to 100 µ*M* protein, which was then passed down a PD-10 desalting column to remove excess reducing agent. Initially, the reduction experiments were performed on the bench (aerobically), but following the observation of rapid oxidation of the haem, the X183 proteins were reduced and passed down the PD-10 column in an anaerobic chamber. Samples thus prepared were sealed using Suba Seals (Sigma–Aldrich) for transfer to the UV–Vis spectrophotometer for measurements.

### 4.5. Thin-film voltammetry using TtX183A, TtX183B and TtX183C

Fourier-transformed large-amplitude AC voltammetry measurements were performed for each of TtX183A, TtX183B and TtX183C using a standard three-electrode setup consisting of a working pyrolytic graphite-edge electrode attached to an Orgiatrod rotator operated in stationary mode, a standard calomel reference electrode and a Pt wire counter electrode. The three electrodes were contained within a custom-built electrochemical cell (constructed by the University of York, Department of Chemistry, Glass Workshop) surrounded by a thermostat-controlled water jacket, which was maintained at 5°C. Each measurement used 10 µl of protein at a concentration of 100 µ*M*, which was pipetted onto a freshly abraded working electrode surface (using emery paper, grade 1200) and left to adsorb for 1 min. The measurements were performed in pH 7.0 buffer consisting of 150 m*M* NaCl and 50 m*M* each of acetate, Tris, phosphate and MES. Measured potentials were converted into values compared with the SHE by the addition of 200 mV.

### 4.6. Expression and purification of Ttpf08450-X173

The coding sequence for TERTU_3803 (GenBank: ACR14707.1) was identified in the *T. turnerae* T7901 genome sequence (NCBI nucleotide CP001614) and was synthesized with codon optimization for expression in *E. coli* by Genewiz. The region encoding Ttpf08450-X173 (nucleotides 1243 to 3204) was subsequently cloned into the pET26b vector downstream of the pelB signal peptide using the PIPE cloning method (Klock & Lesley, 2009 ▸). The protein was produced in BL21(DE3) cells in 1 l 2xYT cultures grown at 37°C, shaking at 180 r min^−1^ until an A_600_ of 0.6 was reached. The culture was then cooled to 20°C and shaken at 120 r min^−1^ before induction with IPTG, which was added to a final concentration of 1 m*M*. The cultures were grown overnight and harvested by centrifugation at 3985*g* for 20 min at 4°C.

The periplasmic lysis was carried out as described above for TtX183A, TtX183B and TtX183C. The protein was purified first by loading onto a 5 ml HisTrap FF (Cytiva) column that had been equilibrated with Buffer A (50 m*M* HEPES pH 7, 200 m*M* NaCl and 30 m*M* imidazole). After washing the column with 5 CVs of Buffer A, the protein was then eluted by applying a linear gradient to give 100% Buffer B (Buffer A + 300 m*M* imidazole) across 20 CVs collecting 1.8 ml fractions. Peak fractions containing Ttpf08450-X173 were combined and concentrated to a volume of ∼5 ml using a 30 kDa molecular-weight cut-off Vivaspin concentrator (Sartorius). The sample was then diluted tenfold using Buffer C (50 m*M* Tris pH 8, 50 m*M* NaCl) and applied to a 5 ml Q FF (Cytiva) column for ion-exchange purification, which had previously been equilibrated in the same buffer. Following sample application, the column was washed with 5 CVs of 50 m*M* Tris pH 8, 50 m*M* NaCl before a linear gradient to 100% Buffer D (Buffer C + 0.5 *M* NaCl) was applied across 20 CVs. Then, 1.8 ml fractions were collected across the gradient. Peak fractions containing Ttpf08450-X173 were once more combined and concentrated using a 30 kDa molecular-weight cut-off concentrator (Sartorius) until the sample had a volume smaller than 1 ml. The sample was then applied to a 16/600 Superdex 200 (Cytiva) gel filtration column, which had been equilibrated in SEC buffer (20 m*M* Tris pH 8, 200 m*M* NaCl). Then, 1.8 ml fractions were collected after the void volume had passed. Peak fractions containing Ttpf08450-X173 were once more combined and concentrated using a 30 kDa molecular-weight cut-off concentrator. The sample was also buffer exchanged with 20 m*M* Tris pH 8 to provide the final sample, which was quantified by A_280_ measurement using an extinction coefficient of 111 870 *M* ^−1^cm^−1^.

### 4.7. d-gluconic acid/d-glucono-δ-lactone assay for Ttpf08450-X173

Using the d-gluconic acid/d-glucono-δ-lactone assay kit (Megazyme), a calibration curve was prepared by serially diluting a 1*M* stock solution of gluconic acid from 1 µ*M* to 200 µ*M* in a final volume of 2.54 ml, using distilled water. Then, 0.1 ml of each dilution was transferred into a cuvette, which contained 2.00 ml of distilled water (at ∼25°C), 0.2 ml of assay buffer (pH 7.6), 0.2 ml of an NADP+ and ATP solution, and 0.02 ml of 6-phospho­gluconate de­hydrogenase (6-PGDH). This sample was mixed by gentle inversion after sealing the cuvette with Parafilm. A blank was also made up, containing 2.10 ml of distilled water, 0.2 ml of buffer, 0.2 ml of the NADP+/ATP solution and 0.02 ml of 6-PGDH. The absorbances of the solutions were read after ∼5 min at 340 nm in a 1 cm light path at ∼25°C in a Cary60 spectrophotometer (Agilent). The reactions in each cuvette (including the blank) were started by the addition of 0.02 ml of gluconate kinase suspension (GCK). The absorbances were taken again after 6 min. These absorbances were plotted to generate a calibration curve.

To test if the pf08450_X173 protein had glucose de­hydrogenase activity, reactions were set up containing 20 m*M* HEPES pH 7.0, 5 m*M* glucose, 1 m*M* PQQ, 2 m*M* CaCl_2_, 1 m*M* ascorbate and ±5 µ*M* Ttpf08450_X173 in a total volume of 0.1 ml. Each reaction was mixed with the assay components in place of the gluconic acid standard described for the generation of the calibration curve. The absorbance of each sample at 340 nm was measured once more after ∼5 min in a Cary60 spectrophotometer (Agilent).

### 4.8. Isothermal titration calorimetry using PQQ and Ttpf08450-X173

ITC experiments were performed with a MicroCal ITC200 instrument (Cytiva). Protein and PQQ samples were prepared in 20 m*M* HEPES pH 7.0, 250 m*M* NaCl. For experiments in the presence of Ca^2+^, 5 m*M* CaCl_2_ was included in the protein and ligand samples. The protein was present in the cell at 20 µ*M* and PQQ was present at 200 µ*M* in the syringe. Titrations were performed at 20°C with a reference power of 5 µcal s^−1^ and a delay between injections of 120 s. The first injection (0.5 µl) was rejected before data analysis.

### 4.9. Expression and purification of TtX122A and TtX122B proteins

Using the synthetic genes that had been ordered for TERTU_3803 and TERTU_2913 described earlier, the coding sequences for TtX122A (nucleotides 370 to 1089 from TERTU_3803) and TtX122B (nucleotides 1042 to 1752 from TERTU_2913) were cloned downstream of the pelB leader peptide in pET26b by the PIPE cloning method (Klock & Lesley, 2009 ▸). Protein was produced from both constructs in BL21(DE3) *E. coli* in 1 l cultures of 2xYT media. Cultures were grown at 37°C with shaking at 200 r min^−1^ until an A_600_ of 0.6 was reached, at which point the temperature was lowered to 16°C, and after 30 min IPTG was added to a final concentration of 1 m*M*. The cultures were incubated overnight before the cells were harvested by centrifugation at 5000*g* for 20 min.

The periplasmic lysis was performed as described for TtX183A, TtX183B and TtX183C above. The proteins were subsequently purified using an analogous protocol to that used for TtX183A, TtX183B and TtX183C. Briefly, the proteins were applied to a 5 ml HisTrap FF (Cytiva) affinity column that had been equilibrated in Buffer A (50 m*M* HEPES pH 7, 200 m*M* NaCl, 30 m*M* imidazole). After washing the column with 5 CVs of Buffer A, a gradient from 0 to 100% Buffer B (Buffer A + 300 m*M* imidazole) was applied across 20 CVs collecting 1.8 ml fractions. Peak fractions were combined and concentrated on a 10 kDa molecular-weight cut-off Vivaspin concentrator (Sartorius) to a volume smaller than 1 ml. The sample was then applied to a 16/600 Superdex 75 (Cytiva) gel filtration column, which had been equilibrated in SEC Buffer 2 (20 m*M* HEPES pH 7, 200 m*M* NaCl). Then, 1.8 ml fractions were collected after a void volume of 40 ml. Peak fractions containing TtX122A or TtX122B were then combined and concentrated on the same 10 kDa molecular-weight cut-off concentrator with buffer exchange into 20 m*M* HEPES pH 7. Samples were quantified from their A_280_ absorbance using the extinction coefficients of 54 555 *M* ^−1^cm^−1^ and 56 045 *M* ^−1^cm^−1^ for TtX122A and TtX122B, respectively.

Se-Met labelled TtX122A was prepared by expressing the protein in the me­thio­nine-auxotrophic *E. coli* strain B834(DE3). The protein was isolated from cells following culturing in M9 minimal media supplemented with BME vitamins (Sigma–Aldrich) and l-(+)-Se-Met (Anatrace), using the same expression protocol and subsequent purification procedure as described above for the native protein. Se-Met incorporation into the protein was confirmed using electrospray-ionization mass-spectrometry analysis and proteins were quantified using the same extinction coefficients as used for the native protein.

### 4.10. Crystallization, X-ray data collection and structure determination for TtX122A and TtX122B

TtX122A was buffer exchanged into 10 m*M* HEPES pH 7 on a protein concentrator following purification and finally concentrated to 18.6 mg ml^−1^ for crystallization trials. Initial crystal hits were obtained in 0.1 *M* magnesium acetate, 20% PEG 3350, which were subsequently optimized in hanging drops by varying the precipitant concentration to yield crystals that were used for data collection and structure determination. Native crystals of TtX122A were transferred into a cryo-protectant solution consisting of the mother liquor supplemented with 20% ethyl­ene glycol. Following a 30 s soak, the samples were then flash frozen for data collection by plunging in liquid nitro­gen. X-ray diffraction data were collected for the native protein on beamline i04 at Diamond Light Source, and were subsequently processed using *XDS* (Kabsch, 2010 ▸) and the *CCP*4*i*2 software suite (Potterton *et al.*, 2018 ▸).

Initial attempts to solve the structure of TtX122A by molecular replacement using the Cip1 structure (PDB ID 3zyp; Jacobson *et al.*, 2013 ▸) as the search template were unsuccessful, so Se-Met labelled TtX122A was prepared as described above. The Se-Met labelled protein crystallized in the same conditions as those used for the native protein and crystals were cryo-protected with mother liquor and 20% ethyl­ene glycol before cryo-cooling for data collection. Diffraction data were collected on beamline i03 of Diamond Light Source at wavelengths of 0.9798, 0.9800 and 0.9645 Å, representing the peak, inflection and high energy remote datasets used for structure determination, respectively. The data were indexed using *XDS* (Kabsch, 2010 ▸) and subsequently processed in the *CCP*4*i*2 software suite (Potterton *et al.*, 2018 ▸). The structure was then determined using the *SHELXC/D/E* pipeline (Sheldrick, 2008 ▸, 2015 ▸) to generate an initial model, which was subsequently built upon manually using successive rounds of rebuilding and refinement in *Coot* (Emsley *et al.*, 2010 ▸) and *REFMAC*5 (Murshudov *et al.*, 1997 ▸), respectively. The crystals of the Se-Met labelled protein were isomorphous to those of native TtX122A, so the model with flexible regions and water molecules removed was rebuilt and refined in *Coot* (Emsley *et al.*, 2010 ▸) and *REFMAC*5 (Murshudov *et al.*, 1997 ▸), respectively, to give the final native TtX122A structure.

TtX122B was buffer exchanged into 10 m*M* HEPES pH 7 on a protein concentrator, as done for TtX122A. Crystals were obtained in sitting drops by mixing equal volumes of protein at 10 mg ml^−1^ with 0.2 *M* NaCl, 25% PEG 3350 and 0.1 *M* bis-Tris pH 6.5. Crystals were harvested directly from the drop without addition of any cryo-protectant and cryo-cooled by direct plunging in liquid nitro­gen. Diffraction data were then collected on beamline i24 of Diamond Light Source at a wavelength of 0.970 Å. The data were indexed using *XDS* (Kabsch, 2010 ▸) and subsequently processed in *CCP*4*i*2 (Potterton *et al.*, 2018 ▸). The structure was determined by molecular replacement in *PHASER* (McCoy *et al.*, 2007 ▸) using the TtX122A protomer structure as the search model. The resulting structure underwent subsequent rounds of manual building and refinement using *Coot* (Emsley *et al.*, 2010 ▸) and *REFMAC*5 (Murshudov *et al.*, 1997 ▸), respectively.

### 4.11. Thin-film liquid chromatography and polysaccharide microarray analysis for TtX122A and TtX122B activity measurements

Initial attempts to detect an enzymatic activity for TtX122A were performed using TLC. First, 2 µ*M* protein was incubated with 2 mg ml^−1^ substrate (Avicel, citrus pectin, alginic acid, polygalacturonic acid, galactomannan, lichenan, rhamnogalacturonan, d-(+)-cellobiose, barley β-glucan, mannan, xyloglucan, wheat arabinoxylan) in 20 m*M* HEPES pH 7.0 in a total volume of 1 ml and left at room temperature overnight, turning end-over-end on a tube rotator (Stuart Scientific). Samples were subsequently centrifuged at 16 000*g* for 2 min to remove any solid material. Then, 100 µl of the supernatant was removed from each sample for analysis. Samples were spotted onto TLC paper, adjacent to a solution of glucose, cellobiose and cellotriose that was used as a standard. The bottom end of the TLC paper was submerged in a solvent of acetic acid and butanol, which was allowed to migrate up the paper until it had reached three quarters of the way up. The TLC paper was subsequently dried using a heat gun and subsequently stained by submerging in a solution of orcinol.

For polysaccharide microarray analysis, defined polysaccharides (Table S3) were dissolved in deionized water to 4 mg ml^−1^, and these were subsequently diluted 40-fold with printing buffer (55.2% glycerol, 44% water, 0.8% Triton X-100). Then, 10 µl of these substrate solutions were added into separate wells of a 384-microwell plate (PP microplate, V-shape, Greiner Bio-One), to which an equal volume of TtX122A or TtX122B at 2 µ*M* concentration was added. Controls were prepared for each substrate solution without enzyme, keeping all other conditions identical. Reactions using *Aspergillus aculeatus* endo-polygalacturonanase M2 (Megazyme) and *Aspergillus* sp. pectate lyase (Megazyme) using polygalacturonate and pectin as substrates were employed as positive controls. The filled plates were incubated at 100 r min^−1^ for 2 h at room temperature and any reaction was then stopped by incubation of the samples at 80°C for 10 min. Insoluble material was pelleted by centrifugation at 5000*g* for 10 min before the soluble material was printed at 22°C and 55% humidity onto a nitro­cellulose membrane with a pore size of 0.45 µm (Whatman) using a Sprint Arrayjet microarray robot (Roslin, UK). The printed arrays were blocked for 1 h in PBS (140 m*M* NaCl, 2.7 m*M* KCl, 10 m*M* Na_2_HPO_4_, 1.7 m*M* KH_2_PO_4_, pH 7.5) with 5%(*w*/*v*) low-fat milk powder (MPBS). Then, the arrays were incubated for 2 h with probes diluted 1:1000 with MPBS, which included polysaccharide-specific mAbs and CBMs (PlantProbes, Leeds, UK; Institut National de la Recherche Agronomique, Nantes, France; BioSupplies, Bundoora, Australia; and NZYTech, Lisbon, Portugal). The arrays were washed thoroughly in PBS and incubated for 2 h with *anti*-rat, *anti*-mouse or *anti*-His tag secondary antibodies conjugated to alkaline phosphatase (Sigma) diluted 1:5000 (*anti*-rat and *anti*-mouse) or 1:1500 (*anti*-His tag) in MPBS. Once washed in PBS and deionized water, microarrays were developed in a solution containing 5-bromo-4-chloro-3-indolylphosphate and nitro blue tetrazolium in alkaline phosphatase buffer (100 m*M* NaCl, 5 m*M* MgCl2, 100 m*M* di­ethano­lamine, pH 9.5) for 10–15 min until spots appeared. Developed arrays were scanned at 2400 dots per inch (CanoScan 8800F, Søborg, Denmark) and converted to TIFFs, followed by probe-signals quantification using *Array-Pro Analyser* 6.3 software (Media Cybernetics, Rockville, Maryland). For analysis, any intensity measurement values less than 5 were given a value of 5, which represented little or no antibody binding. To calculate fold changes in antibody binding between treated samples and the untreated polysaccharides, each spot from the arrays treated with TtX122A/B was divided by the intensity measurements for the no-protein.

### 4.12. Fluorescence microscopy using TtX122:GFP fusion proteins on plant cross sections

Constructs for producing TtX122A and TtX122B fused with GFP were generated by cloning the sfGFP coding sequence into our existing TtX122A and TtX122B pET26b constructs using the PIPE cloning method (Klock & Lesley, 2009 ▸). pET28a-sfGFP [a gift from Ryan Mehl, Addgene plasmid 85492 (Peeler & Mehl, 2012 ▸)] was used as a polymerase chain reaction template to generate the GFP insert. Constructs were designed such that the GFP would be introduced either between the pelB signal peptide and TtX122A/B or between TtX122A/B and the C-terminal His tag, to generate N- and C-terminal GFP fusions, respectively. Additional constructs were also generated in which the CBM10 N-terminal to TtX122A in TERTU_3803 was included to be used as a control. Subsequently, the proteins were produced in BL21(DE3) cells, as described earlier for the TtX122A and TtX122B pET26b constructs, and they were purified in an identical way. The final protein was quantified by A_280_ measurement using the extinction coefficients 73 590 *M* ^−1^cm^−1^, 98 820 *M* ^−1^cm^−1^ and 75 080 *M* ^−1^cm^−1^ for TtX122A:GFP, CBM10-TtX122A:GFP and TtX122B:GFP, respectively.

Tobacco-stem cross sections were prepared and fluorescence microscopy was performed as described by Hervé *et al.* (2010 ▸).

## Supplementary Material

Supporting information. DOI: 10.1107/S2052252524001386/lz5067sup1.pdf

PDB reference: TtX122B, a domain of unknown function from the *Teredinibacter turnerae* protein TERTU_2913, 8q2a

PDB reference: TtX183B , a *c*-type cytochrome domain from the *T. turnerae* protein TERTU_2913, 8q1w

PDB reference: Se-Met labelled TtX122A, a domain of unknown function from the *T. turnerae* protein TERTU_3803, 8q28

PDB reference: TtX183A, a *c*-type cytochrome domain from the *T. turnerae* protein TERTU_2913, 8q1v

PDB reference: TtX122A, a domain of unknown function from the *T. turnerae* protein TERTU_3803, 8q29
