# Supplementary material for: Structural dissection of two redox proteins from the shipworm symbiont Teredinibacter turnerae
Source: IUCrJ. 2024 Mar 1;11(Pt 2):260–74. doi: 10.1107/S2052252524001386 (PMC10916295; doi:10.1107/S2052252524001386)
Supplement: Supplementary file 1 [file m-11-00260-sup1.pdf]

IUCrJ

Volume 11 (2024)

Supporting information for article:

Structural dissection of two redox proteins from the shipworm symbiont *Teredinibacter turnerae*

Badri S. Rajagopal, Nick Yates, Jake Smith, Alessandro Paradisi, Catherine Tétard-Jones, William G.T Willats, Susan Marcus, J. Paul Knox, Mohd Firdaus-Raih, Bernard Henrissat, Gideon J. Davies, Paul H. Walton, Alison Parkin and Glyn R. Hemsworth

Table S1 TtX183A and B Data collection and refinement statistics

	TtX183A	TtX183B
**Data collection**		
Space group	$P4_132$	P1
Cell dimensions		
a, *b*, *c* (Å)	92.7, 92.7, 92.7	38.5, 38.7, 44.0
α, β, γ (°)	90.0, 90.0, 90.0	90.5, 90.6, 94.5
Resolution (Å)	65.53–1.40 (1.42-1.40)	38.54-1.80 (1.84-1.80)
R_{merge}	0.125 (3.874)	0.202 (0.765)
R_{pim}	0.029 (0.894)	0.202 (0.765)
CC(1/2)	1.00 (0.66)	0.82 (0.56)
$I / \sigma I$	22.5 (1.6)	3.4 (1.9)
Completeness (%)	100 (100)	96.8 (95.4)
Multiplicity	36.9 (37.5)	2.3 (2.2)
**Refinement**		
Resolution (Å)	65.61-1.40	38.46-1.80
No. reflections (all/free)	27351/1355	22802/1135
R_{work} / R_{free}	0.164/0.193	0.195/0.240
B-factors (Å^2)		
Protein	19.3	12.4
Ligand	15.2	8.3
Ion/solvent	28.6	9.1
Water	36.2	20.6
R.m.s. deviations		
Bond lengths (Å)	0.017	0.011
Bond angles (°)	2.543	2.400
PDB ID	8q1v	8q1w

*Values in parentheses are for highest-resolution shell.

Table S2 TtX122A and B Data collection and refinement statistics

	Se-TtX122A	TtX122A	TtX122B
**Data collection**			
Wavelength (Å)	†P - 0.9798, I - 0.9800, R - 0.9645	0.9795	0.9686
Space group	$P2_1$	$P2_1$	$P2_12_12_1$
Cell dimensions			
a, *b*, *c* (Å)	48.0, 75.4, 69.8	48.1, 75.2, 69.1	69.1, 75.3, 116.9
α, β, γ (°)	90.0, 107.6, 90.0	90.0, 107.4, 90.0	90.0, 90.0, 90.0
Resolution (Å)	49.88-1.80 (1.84-1.80)	49.55-1.50 (1.53-1.50)	46.69-2.20 (2.27-2.20)
R_{merge}	P - 0.09 (0.28), I - 0.09 (0.29), R - 0.10 (0.31)	0.05 (0.41)	0.37 (2.30)
R_{pim}	P - 0.07 (0.23), I – 0.07 (0.24), R – 0.07 (0.25)	0.04 (0.32)	0.17 (1.07)
CC(1/2)	P - 0.99 (0.91), I – 1.00 (0.94), R – 1.00 (0.92)	1.00 (0.89)	0.60 (0.73)
$I / \sigma I$	P – 20.8 (4.5), I – 20.6 (4.5), R – 19.5 (4.2)	14.5 (2.4)	6.2 (2.9)
Completeness (%)	P – 99.6 (96.1), I – 99.7 (96.0), R – 99.7 (96.4)	98.2 (97.2)	99.8 (98.7)
Multiplicity	P – 6.0 (4.3), I – 5.9 (4.3), R – 6.0 (4.3)	4.5 (4.6)	6.8 (5.6)
**Refinement**			
Resolution (Å)	49.88-1.80	49.60-1.50	46.74-2.20
No. reflections (all/free)	43808/2157	73539/3529	31624/1583
R_{work} / R_{free}	0.157/0.202	0.112/0.156	0.209/0.259
B-factors (Å^2)			
Protein	21.8	20.5	15.8
Ligand	N/A	N/A	N/A
Ion/solvent	31.9	31.1	10.7
Water	30.3	33.8	16.8
R.m.s. deviations			
Bond lengths (Å)	0.016	0.014	0.016
Bond angles (°)	2.02	1.78	2.03
PDB ID	8q28	8q29	8q2a

*Values in parentheses are for highest-resolution shell.

†P = Peak, I = Inflection Point, R = Remote

Table S3 Composition of substrates used in epitope depletion microarrays for TtX122A and B.

**Alginates***	F(G)	F(M)	F(GG)	F(GM)	F(MM)	F(MGG)	F(MGM)	F(GGG)	N(G>1)	Molecular Weight (Da)
PAA	0.8	0.2	0.7	0.1	0.1	0.016	0.094	0.68	44	423767
PAB	0.47	0.53	0	0.47	0.059	0	0.47	0	0	456733
PAC	0.7	0.3	0.55	0.15	0.15	0.083	0.086	0.47	7	262400
PAE	0.52	0.48	0.38	0.14	0.34	0.037	0.12	0.34	11	436767
PAG	0	1	0	0	1	0	0	0	0	584400
PAI	0.32	0.68	0.2	0.12	0.56	0.05	0.07	0.16	6	241100
PAO	0.67	0.33	0.52	0.15	0.18	0.063	0.081	0.46	9	226550
PAT	0.49	0.51	0.33	0.16	0.35	0.04	0.12	0.29	9	260600
PAU	0.17	0.83	0	0.17	0.66	0	0.17	0	0	438733

**Lime Pectin**	**Degree of methyl esterification (DE)**	**Produced by**
B15	15%	removal of ester groups from a high DE pectin (E81) using base
P53	53%	removal of ester groups from a high DE pectin (E81) using a plant derived pectin methyl esterase
B64	64%	removal of ester groups from a high DE pectin (E81) using a base
F11	11%	removal of ester groups from a high DE pectin (E81) using a fungal derived pectin methyl esterase
F43	43%	removal of ester groups from a high DE pectin (E81) using a fungal derived pectin methyl esterase
E81	81%	
P16	16%	removal of ester groups from a high DE pectin (E81) using a plant derived pectin methyl esterase

* Molar ratios of monomers (guluronic acid, G and manuronic acd, M), dimers (GG, GM, and MM) and trimers (MGG, MGM, and GGG)

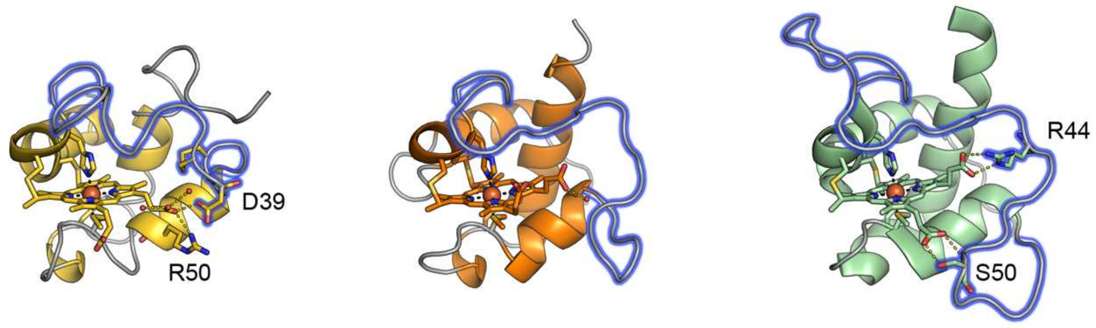

Figure S1 Side by side comparison of the TtX183A (yellow), TtX183B (orange) and CjX183 (green) structures. The loops constituting residues 23-44, 25-48 and 24-56 in TtX183A, TtX183B and CjX183 respectively are highlighted with a blue outline. The interactions between the haem propionate groups and residues within these loops or water molecules are shown by yellow dashed lines. The propionate groups in TtX183A and B form fewer interactions with this loop providing a possible explanation for the lack of stability in the reduced state for these domains compared to CjX183.

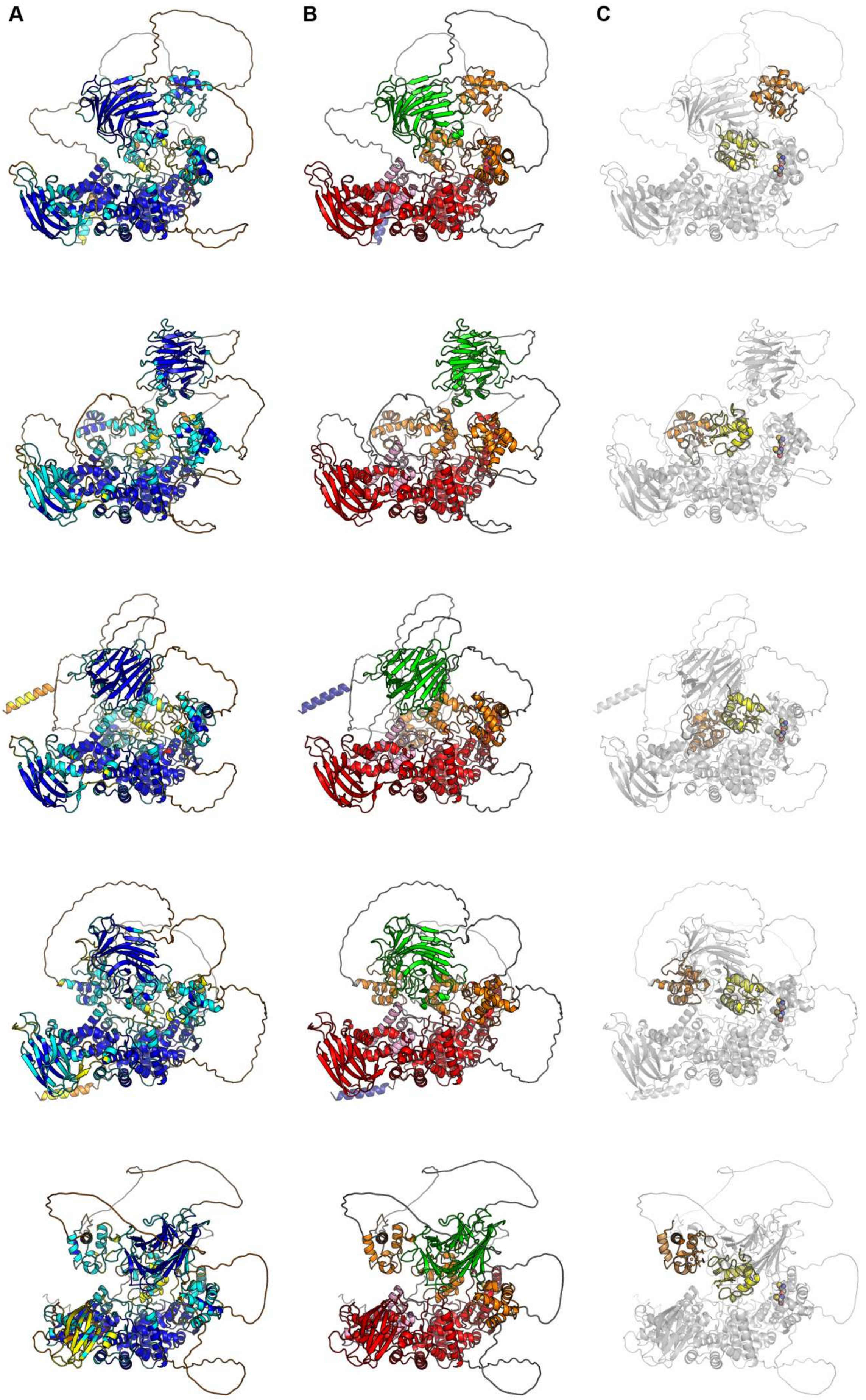
A
B
C

Figure S2 AlphaFold2 Predictions for TERTU_2913. Moving from top to bottom the models are ranked from highest overall pLDDT score to lowest. The models were superposed with one another on the C-terminal X132 domain (residues 843-1242) to allow presentation in the same orientation. **(A)** shows each model coloured by local pLDDT score which determines level of confidence in the local structure prediction. Residues scoring >90 are shown in dark blue, those scoring between 70 and 90 are shown in light blue, residues with scores between 50 and 70 are shown in yellow, and those with scores below 50 are coloured orange. **(B)** shows each model coloured by domain as predicted by CAZy and shown in Figure 1. The signal peptide is coloured dark blue and flexible linkers are coloured grey. The X183 domains are coloured orange, the X122 domain is coloured green, the DUF1687 domain is shown in pink and the X132 domain is shown in red. **(C)** shows the positions of TtX183A (yellow) and TtX183B (orange) following superposition of our experimentally determined structures onto the individual AlphaFold2 models (grey and transparent). The predicted position of the CXXCH motif in the X132 domain is shown by the two cysteine residues shown as spheres in the AlphaFold2 models. From top to bottom TtX183A superposed onto each AlphaFold2 model with rmsd's of 1.576 Å over 74 residues, 1.432 Å over 75 residues, 1.181 Å over 72 residues, 1.344 Å over 72 residues, and 1.543 Å over 72 residues, respectively. For the TtX183B superpositions, the respective rmsd's from top to bottom were 0.590 Å over 79 residues, 0.640 Å over 79 residues, 0.594 Å over 79 residues, 0.743 Å over 79 residues, and 0.906 Å over 73 residues.

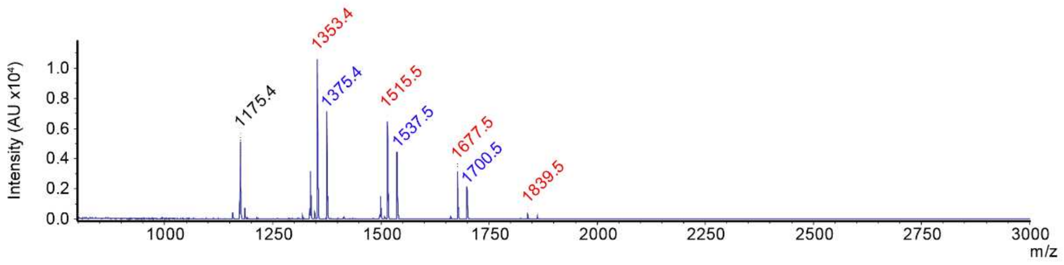

Figure S3 MALDI-ToF analysis of soluble oligosaccharide products following treatment of PASC with TtAA10 and ascorbate. The peaks representing oxidised cello-oligosaccharides are labelled with red and blue labels indicating the masses for the monosodiated and disodiated peaks respectively.

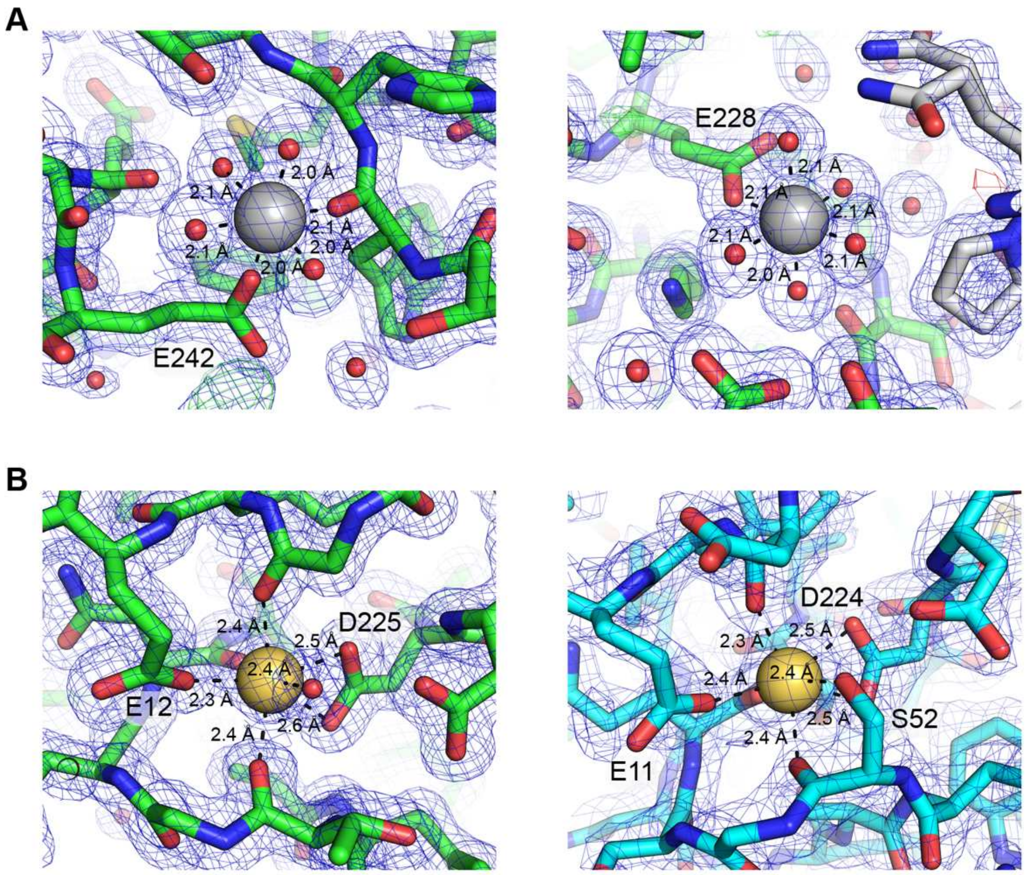

Figure S4 Examples of the electron density observed for **(A)** Mg^{2+} ions present on the surface of TtX122A and **(B)** the Ca^{2+} ion present in TtX122A and TtX122B shown coloured with green and cyan carbon atoms respectively. In all cases the $2F_{obs}$-F_{calc} electron density map is shown contoured at 1σ in blue and the F_{obs}-F_{calc} difference density is shown in green and red for positive and negative electron density contoured at ±4σ respectively.

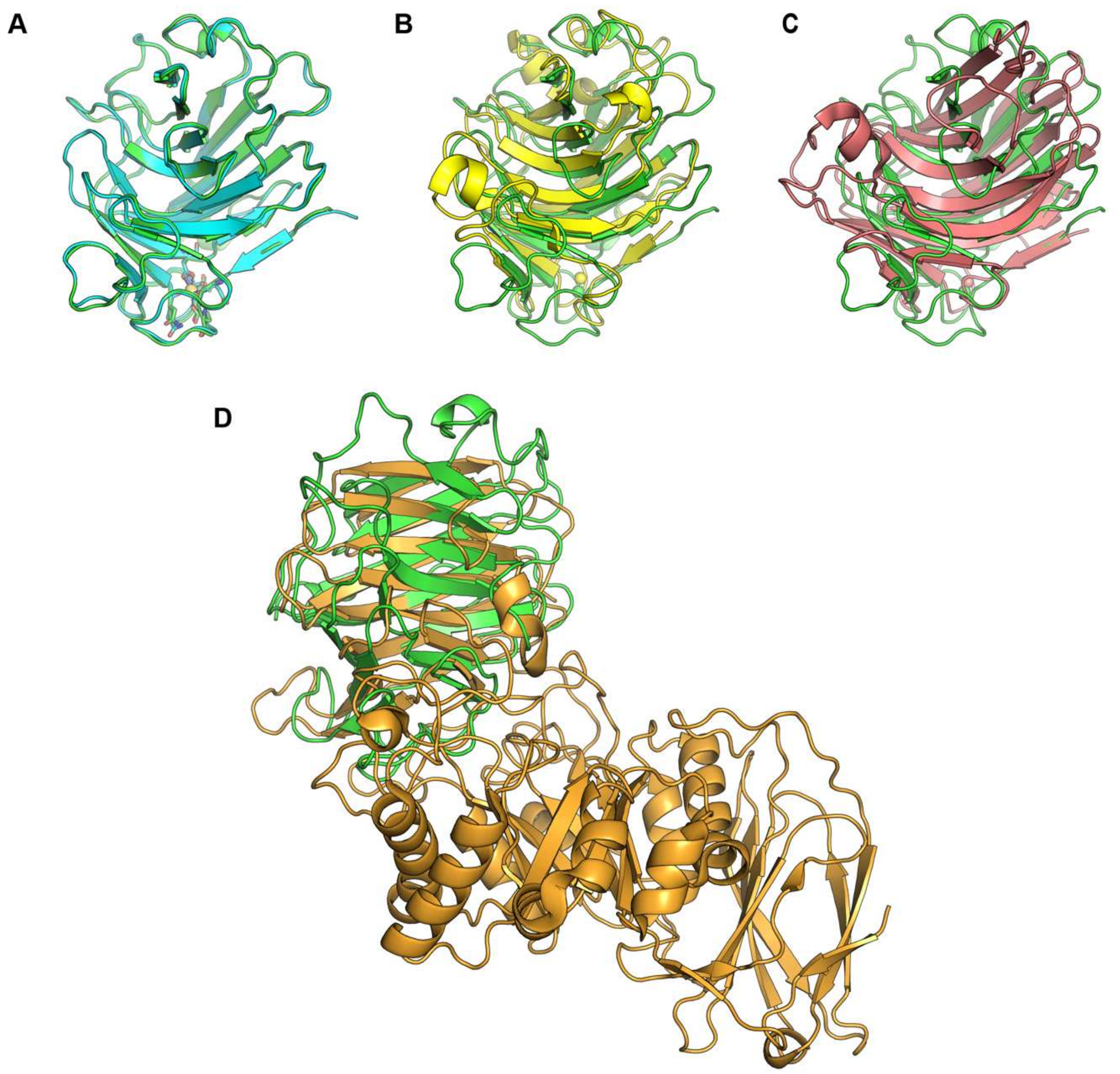

Figure S5 Superpositions of TtX122A (green) with (A) TtX122B (cyan), (B) Cip1 from H. jecorina (yellow, PDBID: 3zyp), (C) TrGL, a PL20 glucuronan lyase from Trichoderma reesei (pink, PDBID: 3zr5) and (D) mouse galactocerebrosidase (orange, PDBID: 5hp6)

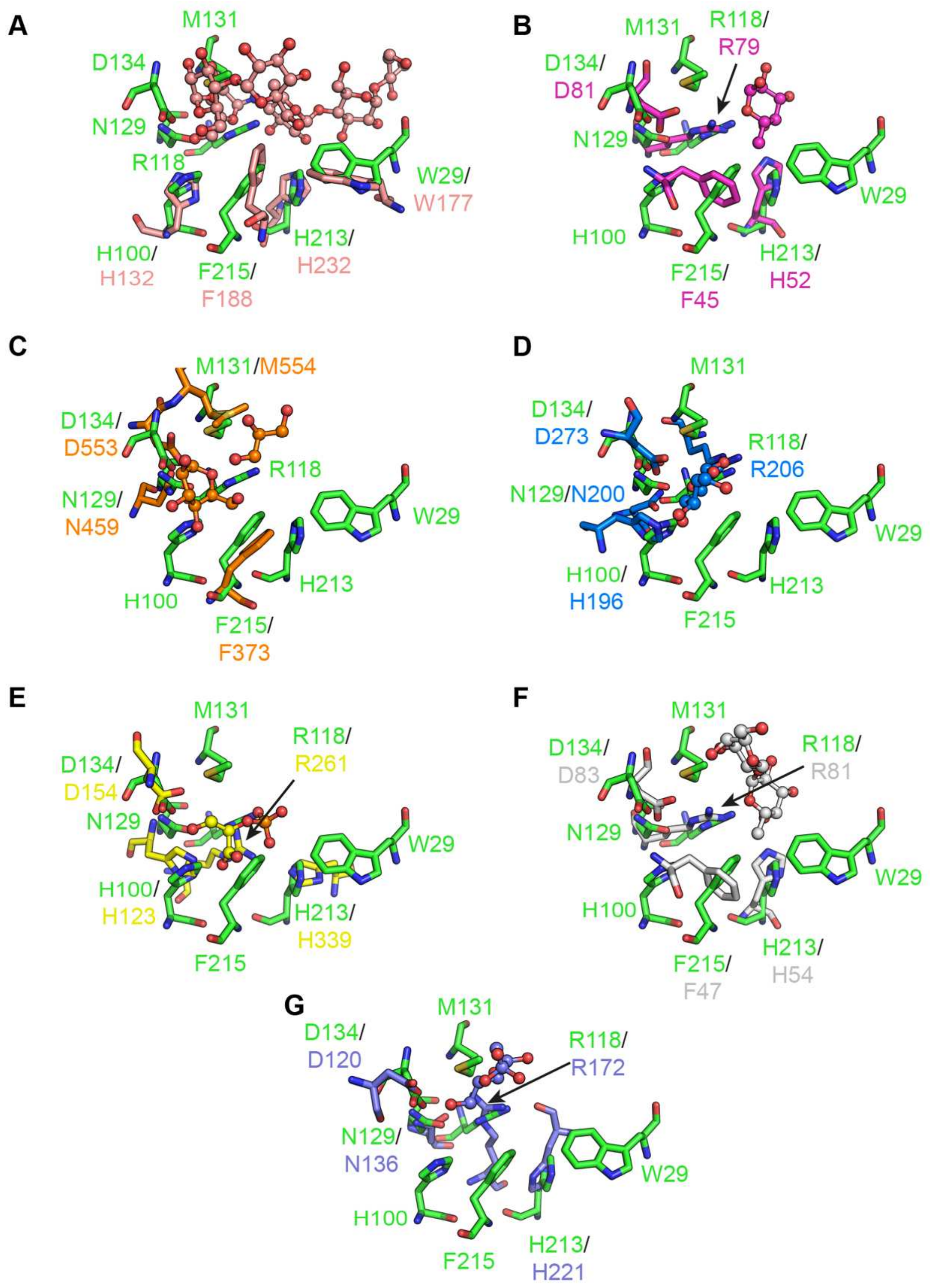

Figure S6 Selected results from the ASSAM analysis using the conserved residues identified in TtX122A as the search pattern. Superpositions with **(A)** an α-amylase from *Bacillus stearothermophilus* in complex with an extended acarbose (pdb id: 1qho, rmsd = 1.21 Å); **(B)** a fucose binding lectin from *Anguilla anguilla* in compelx with fucose (pdb id: 1k12, rmsd = 1.66 Å); **(C)** an α-1,4-glucan lyase from *Gracilariopsis lemaneiformis* with covalent intermediate 5-fluoro-idosyl-fluoride bound (pdb id: 4amw, rmsd – 1.70 Å); **(D)** a β-glucosidase from *Streptomyces venezuelae* in

complex with D-glucose (pdb id: 4i3g, rmsd = 1.67 Å); **(E)** a phosphoglycerate mutase from *Bacillus stearothermophilus* complexed with 2-phosphoglycerate (pdb id: 1o98, rmsd = 1.55 Å); **(F)** A fucose binding lectin *from Streptococcus pneumoniae* in complex with a trisaccharide (pdb id: 2j1v, rmsd = 1.64Å) and **(G)** a fructose binding domain from *Bacteroides thetaiotaomicron* in complex with fructose (pdb id: 2x7x, rmsd = 1.60 Å). In all panels the residues from TtX122A are shown as sticks with carbon atoms coloured green with residues from the superposed structures shown in a contrasting colour. All ligands are shown in ball and stick representation.

TtX122A

	BAM6	BAM7	LM6	LM7	BAM8	LM10	BAM9	LM11	BAM10	LM13	BAM11	LM18	LM2	LM19	LM5	LM21	JIM5	LM22	JIM6	LM23	JIM7	LM24	JIM13	LM25	LM16	LM6-M	C
PAT	1	1	1	1	1	1	1	1	1	1	1	1	1	1	1	1	1	1	1	1	1	1	1	1	1	1	1
PAC	1	1	1	1	2	1	1	1	1	1	1	1	1	1	1	1	1	1	1	1	1	1	1	1	1	1	1
PAG	1	2	1	2	1	1	1	1	2	1	1	1	1	1	1	1	1	1	1	1	1	1	1	1	1	1	1
PAB	1	1	1	1	1	1	1	1	1	1	1	1	1	1	1	1	1	1	1	1	1	1	1	1	1	1	1
PAA	1	2	1	1	1	1	1	1	2	1	1	1	1	1	1	1	1	1	1	1	1	1	1	1	1	1	1
PAE	1	2	1	1	1	1	1	1	1	1	1	1	1	1	1	1	1	1	1	1	1	1	1	1	1	1	1
PAU	1	3	1	2	1	1	1	1	2	1	1	1	1	1	1	1	1	1	1	1	1	1	1	1	1	1	1
PAO	2	2	1	2	2	1	2	1	1	1	1	1	1	1	1	1	1	1	1	1	1	1	1	1	1	1	1
PAI	1	1	1	1	1	1	1	1	1	1	1	1	1	1	1	1	1	1	1	1	1	1	1	1	1	1	1
B15	1	1	1	1	1	1	1	1	1	1	1	1	1	1	1	1	1	1	1	1	1	1	1	1	1	1	1
P53	1	1	1	1	1	1	1	1	1	1	1	1	1	1	1	1	1	1	1	1	1	1	1	1	1	1	1
B64	1	1	1	1	1	1	1	1	1	1	1	1	1	1	1	1	1	1	1	1	1	1	1	1	1	1	1
F11	1	1	1	1	1	1	1	1	1	1	1	1	1	1	1	1	1	1	1	1	1	1	1	1	1	1	1
F43	1	1	1	1	1	1	1	1	1	1	1	1	1	1	1	1	1	1	1	1	1	1	1	1	1	1	1
E81	1	1	1	1	1	1	1	1	1	1	1	1	1	1	1	1	1	1	1	1	1	1	1	1	1	1	1
RG SOY RHAMNOGALCACTURONAN	1	1	1	1	1	1	1	1	1	1	1	1	1	1	1	1	1	1	1	1	1	1	1	1	1	1	1
XYLOGLUCAN	1	1	1	1	1	1	1	1	1	1	1	1	1	1	1	1	1	1	1	1	1	1	1	1	1	1	1
XYLAN	1	1	1	1	1	1	1	1	1	1	1	1	1	1	1	1	1	1	1	1	1	1	1	1	1	1	1
ARABINAN	1	1	1	1	1	1	1	1	1	1	1	1	1	1	1	1	1	1	1	1	1	1	1	1	1	1	1
WHEAT ARABINOXYLAN	1	1	1	1	1	1	1	2	1	1	1	1	1	1	1	1	1	1	1	1	1	1	1	1	1	1	1
RYE ARABINOXYLAN	1	1	1	1	1	1	1	1	1	1	1	1	1	1	2	1	1	1	1	1	1	1	1	1	1	1	1
GALACTAN FROM LUPIN	1	1	1	1	1	1	1	1	1	1	1	1	0	1	1	1	1	1	1	1	1	1	1	0	1	1	1
GALACTOMANNAN	1	1	1	1	1	1	1	1	1	1	1	1	1	1	1	1	1	1	1	1	1	1	1	1	1	1	1
ARABINOXYLAN DEBRANCHED WHEAT	1	1	1	1	1	1	1	1	1	1	1	1	1	1	1	1	1	1	1	1	1	1	1	1	1	1	1
POLYGALACTURONIC ACID	1	1	1	1	1	1	1	1	1	1	1	1	1	1	1	1	1	1	1	1	1	1	1	1	1	1	1
MANNAN	1	1	1	1	1	1	1	1	1	1	1	1	1	1	1	1	1	1	1	1	1	1	1	1	1	1	1
P16	1	1	1	1	1	1	1	1	1	1	1	1	1	1	1	1	1	1	1	1	1	1	1	1	1	1	1

TtX122B

	BAM6	BAM7	LM6	LM7	BAM8	LM10	BAM9	LM11	BAM10	LM13	BAM11	LM18	LM2	LM19	LM5	LM21	JIM5	LM22	JIM6	LM23	JIM7	LM24	JIM13	LM25	LM16	LM6-M	C
PAT	1	2	1	1	1	1	1	1	1	1	1	1	1	1	1	1	1	1	1	1	1	1	1	1	1	1	1
PAC	1	1	1	1	1	1	1	1	1	1	1	1	1	1	1	1	1	1	1	1	1	1	1	1	1	1	1
PAG	2	2	1	1	1	1	1	1	2	1	1	1	1	1	1	1	1	1	1	1	1	1	1	1	1	1	1
PAB	1	1	1	1	1	1	1	1	1	1	1	1	1	1	1	1	1	1	1	1	1	1	1	1	1	1	1
PAA	1	1	1	0	1	1	1	1	1	1	1	1	1	1	1	1	1	1	1	1	1	1	1	1	1	1	1
PAE	1	2	1	1	1	1	1	1	1	1	1	1	1	1	1	1	1	1	1	1	1	1	1	1	1	1	1
PAU	1	1	1	1	1	1	1	1	1	1	1	1	1	1	1	1	1	1	1	1	1	1	1	1	1	1	1
PAO	2	2	1	1	1	1	2	1	1	1	1	1	1	1	1	1	1	1	1	1	1	1	1	1	1	1	1
PAI	1	1	1	1	1	1	1	1	1	1	1	1	1	1	1	1	1	1	1	1	1	1	1	1	1	1	1
B15	1	1	1	1	1	1	1	1	1	1	1	1	1	1	1	1	1	1	1	1	1	1	1	1	1	1	1
P53	1	1	1	1	1	1	1	1	1	1	1	1	1	1	1	1	1	1	1	1	1	1	1	1	1	1	1
B64	1	1	1	1	1	1	1	1	1	1	1	1	1	1	1	1	1	1	1	1	1	1	1	1	1	1	1
F11	1	1	1	1	1	1	1	1	1	1	1	1	1	1	1	1	1	1	1	1	1	1	1	1	1	1	1
F43	1	1	1	1	1	1	1	1	1	1	1	1	1	1	1	1	1	1	1	1	1	1	1	1	1	1	1
E81	1	1	1	1	1	1	1	1	1	1	1	1	1	1	1	1	1	1	1	1	1	1	1	1	1	1	1
RG SOY RHAMNOGALCACTURONAN	1	1	1	1	1	1	1	1	1	1	1	1	1	1	1	1	1	1	1	1	1	1	1	1	1	1	1
XYLOGLUCAN	1	1	1	1	1	1	1	1	1	1	1	1	1	1	1	1	1	1	1	1	1	1	1	1	1	1	1
XYLAN	1	1	1	1	1	1	1	1	1	1	1	1	1	0	1	1	1	1	1	1	1	1	1	1	1	1	1
ARABINAN	1	1	1	1	1	1	1	1	1	1	1	1	1	1	1	1	1	1	1	1	1	1	1	1	1	1	1
WHEAT ARABINOXYLAN	1	1	1	1	1	1	1	3	1	1	1	1	1	1	1	1	1	1	1	1	1	1	1	1	1	1	1
RYE ARABINOXYLAN	1	1	1	1	1	1	1	2	1	1	1	1	1	1	2	1	1	1	1	1	1	1	1	1	1	1	1
GALACTAN FROM LUPIN	1	1	1	1	1	1	1	1	1	1	1	1	1	1	1	1	1	1	1	1	1	1	1	0	1	1	1
GALACTOMANNAN	1	1	1	1	1	1	1	1	1	1	1	1	1	1	1	1	1	1	1	1	1	1	1	1	1	1	1
ARABINOXYLAN DEBRANCHED WHEAT	1	1	1	1	1	1	1	1	1	1	1	1	1	1	1	1	1	1	1	1	1	1	1	1	1	1	1
POLYGALACTURONIC ACID	1	1	1	1	1	1	1	1	1	1	1	1	1	1	1	1	1	1	1	1	1	1	1	1	1	1	1
MANNAN	1	1	1	1	1	1	1	1	1	1	1	1	1	1	1	1	1	1	1	1	1	1	1	1	1	1	1
P16	1	1	1	1	1	1	1	1	1	1	1	1	1	1	1	1	1	1	1	1	1	1	1	1	1	1	1

Positive Controls

	BAM6	BAM7	LM6	LM7	BAM8	LM10	BAM9	LM11	BAM10	LM13	BAM11	LM18	LM2	LM19	LM5	LM21	JIM5	LM22	JIM6	LM23	JIM7	LM24	JIM13	LM25	LM16	LM6-M	C
POLYGALACTURONIC ACID	1	11	8	1	1	1	1	1	1	1	1	10	1	14	6	1	1	1	1	1	1	1	3	9	7	9	1
P16	1	16	1	1	1	1	1	1	1	1	1	13	3	19	13	2	10	1	1	1	16	1	8	11	12	1	1

Figure S7 Epitope depletion microarray screening for TtX122A and B. The magnitude of the binding change for antibodies (shown along the top) to each substrate (shown along the side) are indicated by the values that represent the fold change of binding. Loss of antibody binding is indicated by the intensity of the green colour with little or no change shown in yellow. See Table S3 for explanation of substrate compositions relating to the alginates and pectins that were screened.

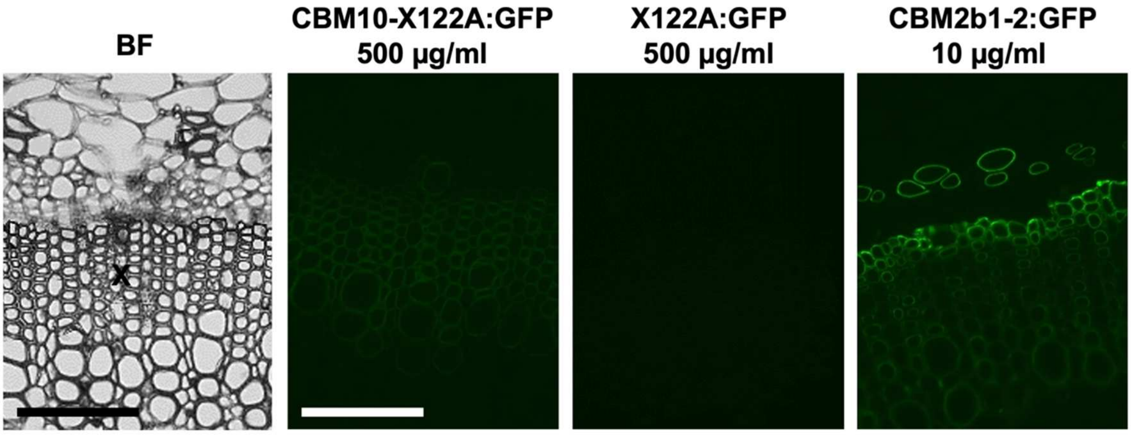

Figure S8 Representative data for the direct fluorescence probing of transverse sections of tobacco stem with GFP fusion proteins. CBM10-X122A:GFP and X122A:GFP are shown alongside a positive control of the xylan-directed CBM2b1-2:GFP. There was no evidence of binding for the X122A protein when applied at 500 µg/ml and a very weak signal for the CBM10-X122A construct. Fluorescence signal for CBM2b2-2:GFP applied at 10 µg/ml shown for comparison. All proteins incubated on sections for at least 1 h. Bright field (BF) image of equivalent region of a stem section. x, xylem. Scale bars: 100 µm.
